# ASCT2 is a major contributor to serine uptake in cancer cells

**DOI:** 10.1016/j.celrep.2024.114552

**Published:** 2024-07-27

**Authors:** Kelly O. Conger, Christopher Chidley, Mete Emir Ozgurses, Huiping Zhao, Yumi Kim, Svetlana E. Semina, Philippa Burns, Vipin Rawat, Lina Lietuvninkas, Ryan Sheldon, Issam Ben-Sahra, Jonna Frasor, Peter K. Sorger, Gina M. DeNicola, Jonathan L. Coloff

**Affiliations:** 1 Department of Physiology and Biophysics, University of Illinois Cancer Center, University of Illinois College of Medicine, Chicago, IL, USA; 2 Laboratory of Systems Pharmacology, Harvard Program in Therapeutic Science, Harvard Medical School, Boston, MA, USA; 3 Department of Cancer Metabolism and Physiology, H. Lee. Moffitt Cancer Center, Tampa, FL, USA; 4 Metabolic and Nutritional Programming, Center for Cancer and Cell Biology, Van Andel Institute, Grand Rapids, MI, USA; 5 Robert H. Lurie Cancer Center, Department of Biochemistry and Molecular Genetics, Northwestern University, Chicago, IL, USA; 6 Department of Systems Biology, Harvard Medical School, Boston, MA, USA; 7 Lead contact

## Abstract

The non-essential amino acid serine is a critical nutrient for cancer cells due to its diverse biosynthetic functions. While some tumors can synthesize serine *de novo*, others are auxotrophic and therefore reliant on serine uptake. Importantly, despite several transporters being known to be capable of transporting serine, the transporters that mediate serine uptake in cancer cells are not known. Here, we characterize the amino acid transporter ASCT2 (*SLC1A5*) as a major contributor to serine uptake in cancer cells. ASCT2 is well known as a glutamine transporter in cancer, and our work demonstrates that serine and glutamine compete for uptake through ASCT2. We further show that ASCT2-mediated serine uptake is essential for purine nucleotide biosynthesis and that estrogen receptor α (ERα) promotes serine uptake by directly activating *SLC1A5* transcription. Collectively, our work defines an additional important role for ASCT2 as a serine transporter in cancer and evaluates ASCT2 as a potential therapeutic target.

## In brief

Despite significant interest in targeting serine metabolism for cancer therapy, the transporters that mediate serine uptake in cancer cells are not known. Conger et al. describe the amino acid transporter ASCT2 as a major contributor to serine uptake in cancer cells that is required for tumor growth in low-serine conditions.

## Graphical Abstract

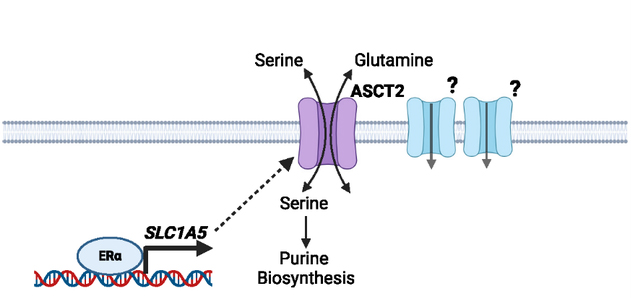

## INTRODUCTION

Amino acid metabolism is central to a cell’s ability to accumulate biomass and proliferate.^1,2^ In particular, the non-essential amino acid L-serine (serine) has received considerable attention, in part because of its importance as a precursor for the synthesis of numerous macromolecules including proteins, lipids, and nucleotides.^3–8^ As a non-essential amino acid, serine can be synthesized via the serine synthesis pathway or consumed through dietary protein.^6^ It has been observed that some cancer cells increase serine synthesis pathway flux by increasing expression of the synthesis pathway genes *PHGDH*, *PSAT1*, and *PSPH*.^9,10^ These and other observations motivated the development of PHGDH inhibitors as potential cancer therapies.^11^ However, in most cases uptake of exogenous serine appears to be sufficient to offset inhibition of the synthesis pathway,^12,13^ thus limiting the efficacy of PHGDH inhibitors as potential therapeutic agents.

The importance of exogenous serine uptake has prompted investigations into serine starvation as an alternative approach to targeting serine metabolism in cancer.^14^ Deprivation of serine can be achieved *in vivo* through dietary serine and glycine restriction, which reduces circulating serine levels by ~50%.^15–17^ Previous work has identified several genetic alternations that increase sensitivity to dietary serine starvation,^15–17^ as well as how dietary serine deprivation can sensitize cancer cells to other therapies.^16,18,19^ Importantly, cancer cells that are auxotrophic for serine are particularly sensitive to exogenous serine starvation both *in vitro* and *in vivo*.^17,20^ Tumors that are naturally auxotrophic for serine, including luminal/estrogen-receptor-positive (ER^+^) breast tumors,^20^ are prime candidates for serine deprivation therapy.^17,21–23^ While dietary serine starvation is a promising therapeutic approach, long-term systemic serine deficiency has the potential to cause peripheral neuropathy.^24–29^ It has also recently been observed that stromal cells in the tumor microenvironment can secrete serine for uptake by auxotrophic cancer cells, which could also limit the efficacy of serine starvation therapy.^23^

Another potential approach of starving cancer cells of serine that could be used alone or in combination with serine starvation is preventing serine uptake into cancer cells. However, the transporters that mediate serine uptake in cancer are largely unknown. Several transporters belonging to the SLC superfamily of proteins facilitate amino acid uptake and maintain cellular amino acid homeostasis.^30^ Initial *in vitro* characterization of substrate specificity for amino acid transporters using proteoliposomes and *Xenopus laevis* oocytes has identified six transporters—ASCT1 (*SLC1A4*),^31^ ASCT2 (*SLC1A5*),^32–35^ SNAT1 (*SLC38A1*),^36,37^ SNAT2 (*SLC38A2*),^33,38^ LAT1 (*SLC7A5*),^33,39^ and LAT2 (*SLC7A8*)^40^—as being capable of transporting serine. ASCT1 acts as a serine transporter in the brain,^41–44^ and ASCT1 and ASCT2 have been shown to promote serine uptake in human cells,^45^ but characterization of serine transporters in cancer cells is lacking. Recently, SFXN1 has been characterized for its contributions to mitochondrial serine transport in leukemia cells,^46^ but it is unlikely to contribute to extracellular serine uptake due to its localization at the mitochondria.

Here, we present evidence that the amino acid transporter ASCT2 (coded by the gene *SLC1A5*) is a major contributor to serine uptake in luminal/ER^+^ breast cancer and other tumor cell lines. While ASCT2 has been extensively studied as a glutamine transporter in cancer and has been shown to be capable of transporting serine in *in vitro* assays,^34,35,47–50^ our work is the first to describe a fundamental role for ASCT2 in cancer cell serine metabolism. Although ASCT2 contributes to serine uptake in all cancer cell lines tested, we find that ASCT2-mediated serine uptake is particularly important in serine-auxotrophic cells and in limited-serine conditions. Additionally, we demonstrate that serine provided by ASCT2 is critical for purine nucleotide biosynthesis and characterize a mechanism of *SLC1A5* regulation by estrogen receptor α (ERα) in ER^+^ breast cancer. Finally, we establish that ASCT2 may be a viable therapeutic target in combination with dietary serine starvation in luminal breast cancer. This work expands our understanding of serine metabolism and amino acid transporter biology in cancer and has important implications in the effort to target serine metabolism for cancer therapy.

## RESULTS

### Identification of ASCT2 as a serine transporter in luminal/ER^+^ breast cancer cells

**Figure 1.**
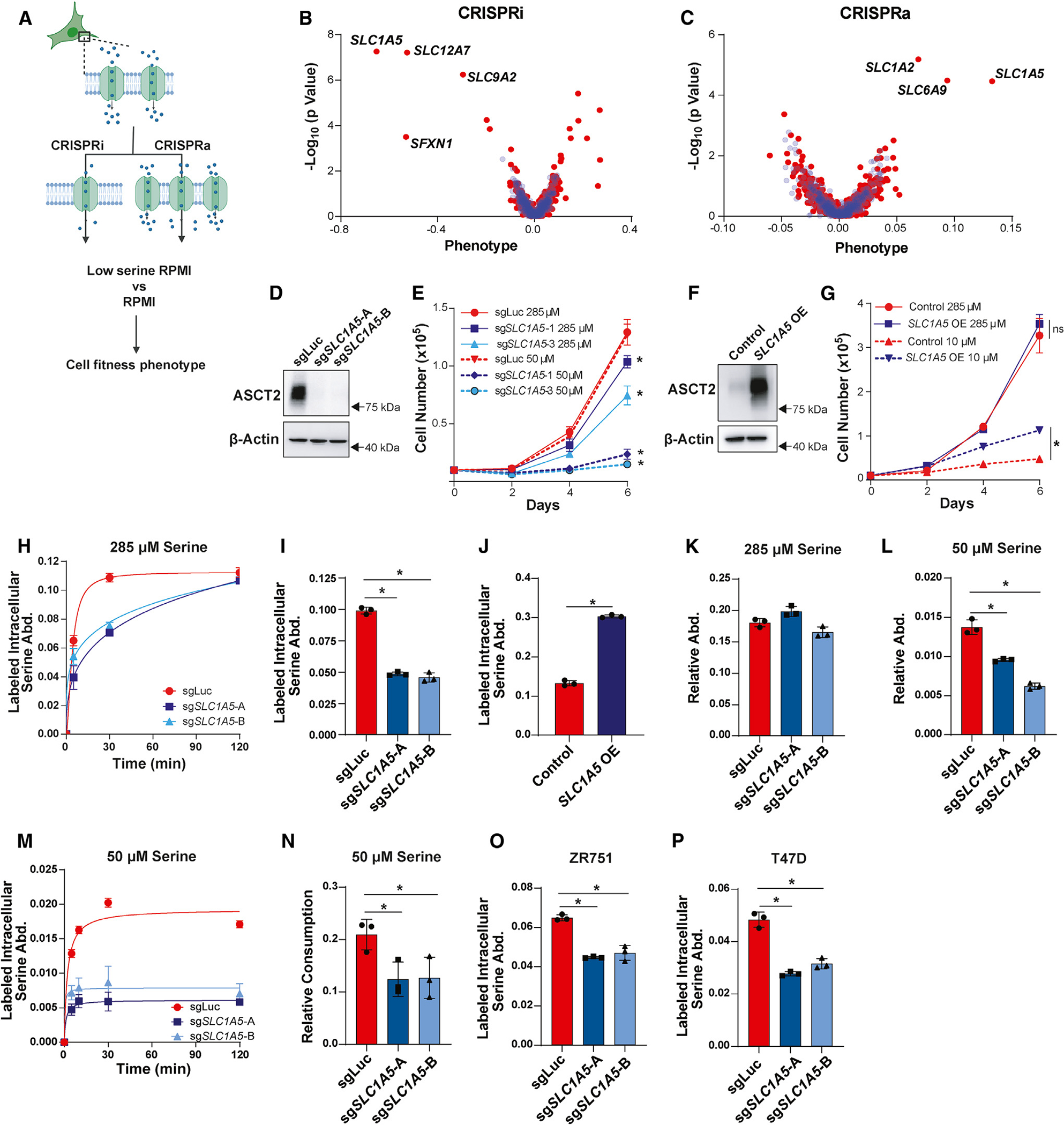
Identification of ASCT2 as a serine transporter in luminal breast cancer cells (A) Schematic of the CRISPRi/CRISPRa screening method. (B and C) Results of the CRISPRi (B) and CRISPRa (C) screen performed in RPMI with low serine (50 μM for CRISPRi, 19 μM for CRISPRa). Blue dots are pseudogenes that represent the technical noise of the assay, and red dots represent transporter genes. (D) Representative western blot of control (sgLuc) and ASCT2 KO (sg*SLC1A5*-A and sg*SLC1A5*-B) MCF7 cells. (E) Growth assay of MCF7 control (sgLuc) or ASCT2 KO (sg*SLC1A5*) cells in complete (285 μM serine) RPMI (solid lines) or low-serine (50 μM serine) RPMI (dotted lines). Values are the mean ± SD of triplicate samples from an experiment representative of three independent experiments. *p < 0.05 by two-way ANOVA. MCF7 sgLuc is compared to sg*SLC1A5* for each serine dose. (F) Representative western blot of control and ASCT2 overexpressing (*SLC1A5* OE) MCF7 cells. (G) Growth assay of control and ASCT2 overexpressing (*SLC1A5* OE) MCF7 cells in complete (285 μM serine) RPMI (solid lines) or low-serine (10 μM serine) RPMI (dotted lines). Values are the mean ± SD of triplicate samples. *p < 0.05 by two-way ANOVA. (H) Accumulation of U-^13^C_3_-^15^N-serine in MCF7 sgLuc and sg*SLC1A5* cells over time in complete media. Values are the mean ± SD of triplicate samples from an experiment representative of two independent experiments. (I) Acute serine uptake in MCF7 control (sgLuc) and ASCT2 KO (sg*SLC1A5*) cells in complete media. Values are the mean ± SD of triplicate samples from an experiment representative of three independent experiments. *p < 0.05 by Welch’s t test. (J) Acute serine uptake in MCF7 control and ASCT2 overexpressing (*SLC1A5* OE) cells in complete media. Values are the mean ± SD of triplicate samples from an experiment representative of three independent experiments. *p < 0.05 by Welch’s t test. (K) Intracellular serine abundance in MCF7 control (sgLuc) and ASCT2 KO (sg*SLC1A5*) cells cultured in complete media. Values are the mean ± SD of triplicate samples from an experiment representative of two independent experiments. (L) Intracellular serine abundance in MCF7 control (sgLuc) and ASCT2 KO (sg*SLC1A5*) cells after exposure to low-serine (50 μM) conditions for 6 h. Values are the mean ± SD of triplicate samples from an experiment representative of two independent experiments. *p < 0.05 by Welch’s t test. (M) Accumulation of U-^13^C_3_-^15^N-serine in MCF7 sgLuc and sg*SLC1A5* cells after exposure to low-serine (50 μM) conditions for 6 h. Values are the mean ± SD of triplicate samples. (N) Net consumption of serine from the medium by MCF7 control (sgLuc) and ASCT2 KO (sg*SLC1A5*) cells after exposure to low-serine (50 μM) conditions for 6 h. Values are the mean ± SD of triplicate samples from an experiment representative of two independent experiments. *p < 0.05 by Welch’s t test. (O) Acute serine uptake in ZR751 control and ASCT2 KO (sg*SLC1A5*) cells in complete media. Values are the mean ± SD of triplicate samples from an experiment representative of three independent experiments. *p < 0.05 by Welch’s t test. (P) Acute serine uptake in T47D control and KO (sg*SLC1A5*) cells in complete media. Values are the mean ± SD of triplicate samples from an experiment representative of three independent experiments. *p < 0.05 by Welch’s t test.

We have recently discovered that luminal/ER^+^ breast cancer cells are auxotrophic for serine^20^ and reasoned that their dependence on exogenous serine would make them an ideal model for identifying serine transporters. Because there are numerous transporters that have the potential to transport serine, we made use of a two-part CRISPRi and CRISPRa screen with a library targeting 489 human transporter genes belonging to the SLC and ABC families.^51^ This library has recently been used to identify transporters for a variety of amino acids, but these efforts were less successful for identifying serine transporters given that they were screened in cells that are not auxotrophic for serine.^51^ Using this library, we therefore utilized our pooled screening approach to construct single-transporter knockdown or overexpression cells in the serine-auxotrophic luminal/ER^+^ breast cancer cell line MCF7. To focus our screen on serine transporters and not just essential transporter genes, we compared the impact of SLC and ABC gene perturbation in RPMI-1640 (RPMI) medium with normal serine levels (285 μM) and low serine levels (50 μM for CRISPRi and 19 μM for CRISPRa). These concentrations were determined in pre-screen titrations and chosen to achieve selective pressure favoring the isolation of hypersensitive hits in CRISPRi screen and resistant hits in CRISPRa screen. This strategy allows the identification of transporters that contribute to serine uptake in MCF7 cells at endogenous expression levels (CRISPRi) and also of transporters that have the ability to transport serine when overexpressed (CRISPRa) (Figure 1A). These low serine concentrations led to 24% and 81% reductions in population doublings during the CRISPRi and CRISPRa screens, respectively (data not shown).

Using this approach, we identified *SLC1A5*, which codes for the amino acid transporter ASCT2, as the top-scoring hit in both the CRISPRi and CRISPRa screens (Figures 1B and 1C). Another significant hit in our CRISPRi screen was *SFXN1*, which was recently shown to be a mitochondrial serine transporter,^46^ demonstrating that our screening approach is capable of identifying transporters involved in serine metabolism. *SLC12A7*^52^ and *SLC9A2*^53^ also scored in our CRISPRi screen (Figure 1B), but their specificity as ion transporters suggests that their phenotypes likely arise via indirect effects on serine metabolism. *SLC1A2* (glutamate)^54^ and *SLC6A9* (glycine)^55^ were also hits in our CRISPRa screen (Figure 1C), but it is unclear whether these transporters directly contribute to serine uptake or rather provide other amino acids that support growth in low-serine conditions.

To validate ASCT2’s role as a serine transporter, we constructed *SLC1A5* MCF7 knockout (KO) cells using CRISPR-Cas9 and two unique single-guide RNAs (sgRNAs) (Figure 1D), whereby we found that loss of ASCT2 slightly but significantly reduces cell growth in normal RPMI (285 μM serine) but strongly reduces proliferation when cultured in the low serine concentration used for the CRISPRi screen (50 μM) (Figures 1E, S1A, and S1B). Conversely, overexpression of ASCT2 significantly rescues growth in very low serine concentration (10 μM), where control cell growth is severely inhibited (Figures 1F, 1G, and S1C). To directly test whether loss of ASCT2 affects serine uptake, we treated control and KO cell lines with media containing U-^13^C_3_-^15^N-serine and measured the accumulation of intracellular labeled serine over time by gas chromatography-mass spectrometry (GC-MS), which revealed a significant delay in serine uptake in ASCT2 KO cells that caught up to control cells after 2 h (Figure 1H). Because intracellular labeled serine plateaued by 30 min in control cells (Figure 1H), we measured intracellular labeled serine abundance at a shorter, acute time point of 4 min. This approach revealed that ASCT2 KO cells exhibit decreased acute serine uptake (Figure 1I), while overexpression of ASCT2 significantly increases serine uptake in MCF7 cells (Figure 1J). Notably, we did not observe differences in total serine abundance in ASCT2 KO cells in full-serine conditions (Figure 1K); however, ASCT2 KO cells display decreased serine abundance after 6 h of culture at low serine (50 μM) (Figure 1L). Accordingly, we found that ASCT2 KO cells cultured at low serine have a severely depleted ability to take up serine that is not able reach the levels of controls cells even after 2 h (Figure 1M). ASCT2 KO cells also consume less serine (as measured by serine depletion from the medium) when cultured in 50 μM serine (Figure 1N). Knockout of ASCT2 in two other luminal breast cancer cell lines revealed a similar decrease in acute serine uptake (Figures 1O, 1P, S1D, and S1E). Together, these results demonstrate that ASCT2 plays a direct role in serine uptake in luminal breast cancer cells and is particularly important in low serine concentrations.

### ASCT2 contributes to serine uptake but does not sensitize to low serine in non-auxotrophic cells

**Figure 2.**
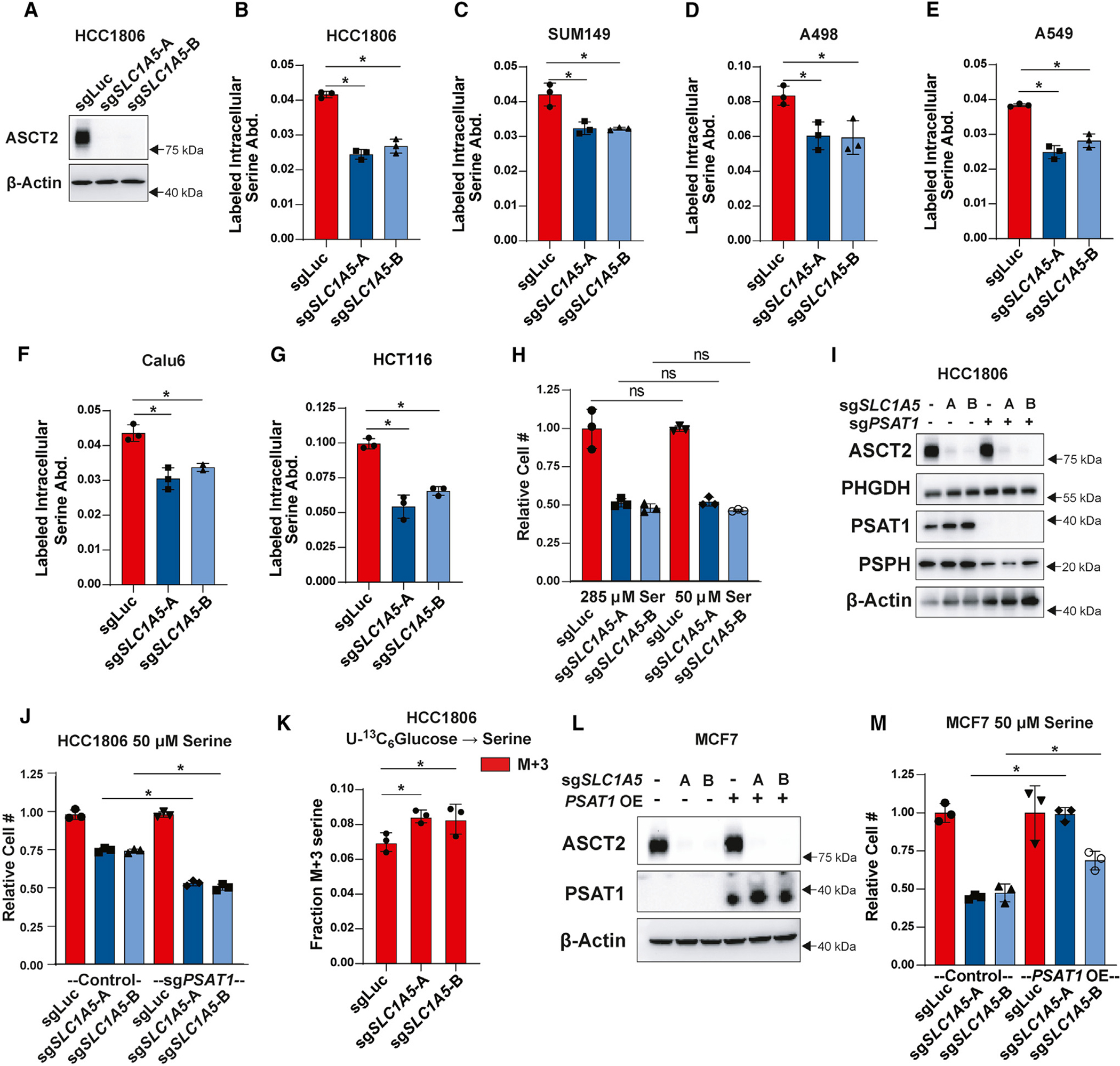
ASCT2 contributes to serine uptake in non-auxotrophic cells (A) Representative western blot of control (sgLuc) and ASCT2 KO (sg*SLC1A5*-A and sg*SLC1A5*-B) HCC1806 cells. (B) Acute serine uptake in HCC1806 control (sgLuc) and ASCT2 KO (sg*SLC1A5*) in complete media. Values are the mean ± SD of triplicate samples from an experiment representative of three independent experiments. *p < 0.05 by Welch’s t test. (C–G) Acute serine uptake in SUM149 (C), A498 (D), A549 (E), Calu6 (F), and HCT116 (G) control (sgLuc) and ASCT2 KO (sg*SLC1A5*) cell lines at complete RPMI concentration (285 μM serine). Values are the mean ± SD of triplicate samples from an experiment representative of three independent experiments. *p < 0.05 by Welch’s t test. (H) Growth assay of HCC1806 control (sgLuc) or ASCT2 KO (sg*SLC1A5*) cells grown in complete RPMI (285 μM) or low-serine (50 μM) RPMI. Values are the mean ± SD of triplicate samples from an experiment representative of three independent experiments. ns, not significant by Welch’s t test. (I) Representative western blot of HCC1806 with either ASCT2 KO alone (sg*SLC1A5*) or in combination with KO of PSAT1 (sg*PSAT1*). (J) Growth assay of HCC1806 ASCT2 KO alone and ASCT2/PSAT1 double KO cells grown in low-serine (50 μM) RPMI. Values are the mean ± SD of triplicate samples from an experiment representative of three independent experiments. *p < 0.05 by Welch’s t test. (K) *De novo* serine biosynthesis from U-^13^C_6_ glucose in HCC1806 control (sgLuc) and ASCT2 KO (sg*SLC1A5*) cells for 24 h. Values are the mean ± SD of M+3-labeled serine from triplicate samples from an experiment representative of two independent experiments. *p < 0.05 by Welch’s t test. (L) Representative western blot of MCF7 cells with either ASCT2 KO alone or in combination with PSAT1 overexpression. (M) Growth assay in low-serine (50 μM) RPMI of MCF7 ASCT2 single KO, PSAT1 OE, and combined ASCT2 KO/PSAT1 OE. Values are the mean ± SD of triplicate samples from an experiment representative of two independent experiments. *p < 0.05 by Welch’s t test.

While serine-auxotrophic cell lines require uptake of exogenous serine, non-auxotrophic cells can synthesize serine *de novo* and are less sensitive to low-serine conditions.^20^ To determine whether ASCT2 contributes to serine uptake in non-auxotrophic cell lines, we knocked out ASCT2 in the basal/triple-negative breast cancer cell lines HCC1806 and SUM149 that we have previously shown to be non-auxotrophic for serine^20^ (Figures 2A and S2A). Similar to the luminal/ER^+^ lines, we found that HCC1806 and SUM149 cells lacking ASCT2 display decreased serine uptake (Figures 2B and 2C). We also knocked out ASCT2 in A498 clear cell renal cell carcinoma (ccRCC) cells, A549 and Calu6 lung cancer cell lines, and HCT116 colorectal cancer cell line and observed decreased serine uptake in all cases (Figures 2D–2G and S2B–S2E), demonstrating that ASCT2 contributes to serine uptake even in non-auxotrophic cell lines and cell lines from diverse tumor types. While loss of ASCT2 does reduce proliferation of HCC1806 cells in full-serine conditions (likely due to reduced glutamine uptake^47^), it does not sensitize to low serine (Figure 2H). Importantly, however, preventing *de novo* serine biosynthesis by knocking out *PSAT1*^20^ does sensitize HCC1806 cells to loss of ASCT2 in low-serine conditions (Figures 2I and 2J). Additionally, loss of ASCT2 in HCC1806 cells also leads to increased serine biosynthesis from glucose (Figure 2K), further indicating the importance of ASCT2 in serine metabolism. Finally, overexpression of PSAT1 in MCF7 cells partially rescues growth in low serine after loss of ASCT2 (Figures 2L and 2M). Together, these data suggest that ASCT2 acts as a serine transporter in both auxotrophic and non-auxotrophic cell lines, but only auxotrophic cells rely on ASCT2 to support growth in low-serine conditions.

### Transporter compensation in the absence of ASCT2

**Figure 3.**
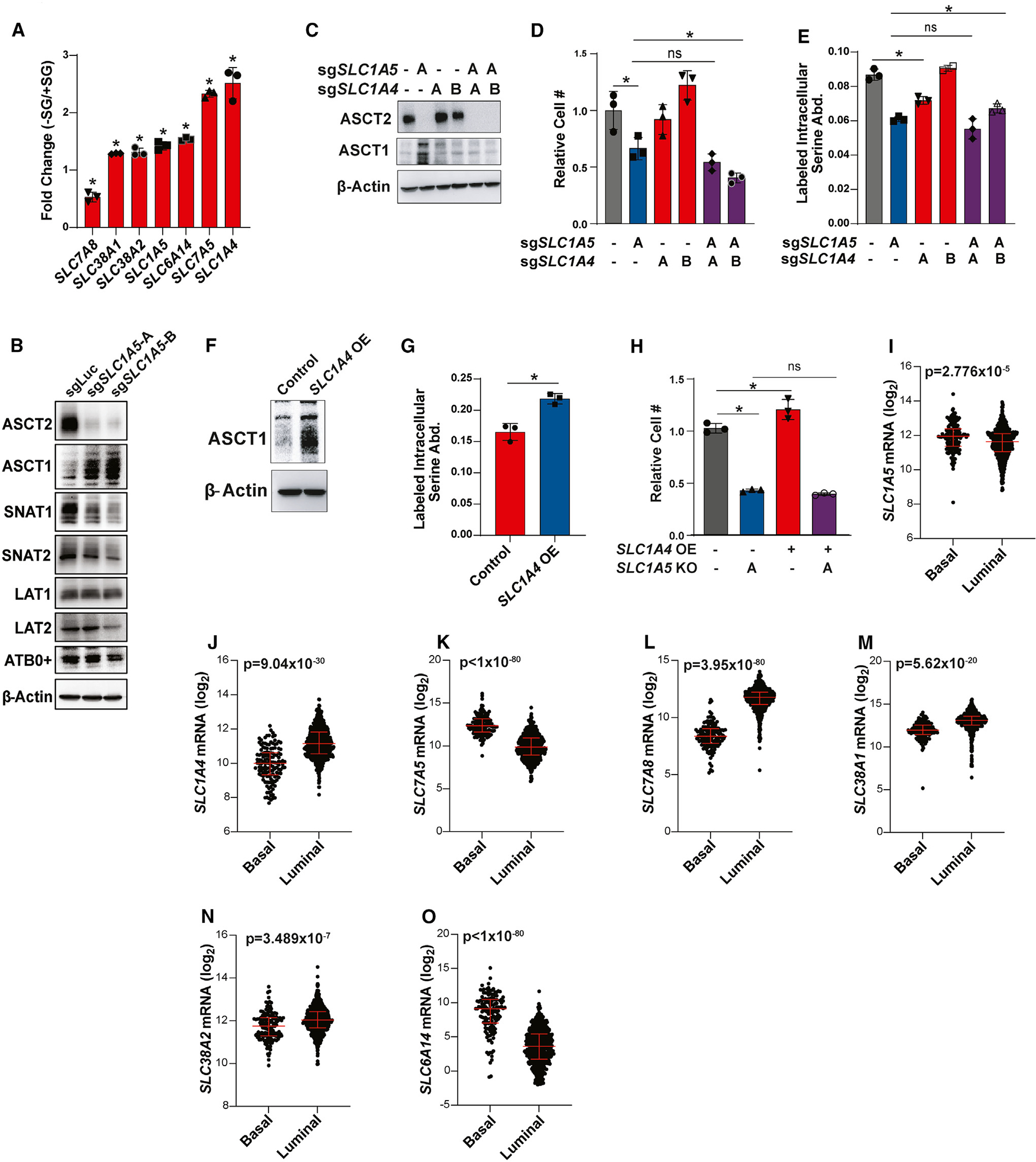
Transporter compensation in the absence of ASCT2 (A) RNA-seq from MCF7 cells grown in the presence or absence of serine and glycine for 48 h prior to harvesting RNA. Values represent the fold change difference in −SG vs. +SG conditions for triplicate samples. *p < 0.05 by Welch’s t test comparing −S/G to +S/G conditions. (B) Western blot from MCF7 control (sgLuc) and ASCT2 KO (sg*SLC1A5*) cells. (C) Representative western blot from single and double ASCT1 (sg*SLC1A4*) and ASCT2 (sg*SLC1A5*) KO MCF7 cells. (D) Growth assay in low (50 μM) serine for single and double ASCT1 (sg*SLC1A4*) and ASCT2 (sg*SLC1A5*) KO MCF7 cells. Values are the mean ± SD of triplicate samples from an experiment representative of two independent experiments. *p < 0.05 by Welch’s t test. ns, not significant. (E) Acute serine uptake assay from single and double ASCT1 (sg*SLC1A4*) and ASCT2 (sg*SLC1A5*) KO MCF7 cells. Values are the mean ± SD of triplicate samples from an experiment representative of two independent experiments. *p < 0.05 by Welch’s t test. ns, not significant. (F) Representative western blot for control and ASCT1 overexpressing (*SLC1A4* OE) MCF7 cells. (G) Acute serine uptake from control and ASCT1 overexpressing (*SLC1A4* OE) MCF7 cells in complete RPMI medium. Values are the mean ± SD of triplicate samples from an experiment representative of two independent experiments. *p < 0.05 by Welch’s t test. (H) Growth assay in ASCT2 KO (sg*SLC1A5*) MCF7 cells with or without ASCT1 overexpression (*SLC1A4* OE) grown at low (50 μM) serine. Values are the mean ± SD of triplicate samples from an experiment representative of three independent experiments. *p < 0.05 by Welch’s t test. ns, not significant. (I–O) Transporter gene expression by breast tumor subtype in The Cancer Gemome Atlas Pan-Cancer Atlas dataset.

Loss of ASCT2 reduces but does not eliminate serine uptake, suggesting that there are additional transporters that also contribute to serine uptake. We interrogated potential secondary transporters based on those published as capable of transporting serine: ASCT1 (*SLC1A4*),^31,41–45^ SNAT1 (*SLC38A1*),^36,37^ SNAT2 (*SLC38A2*),^33,38,56^ LAT1 (*SLC7A5*),^33,39^ LAT2 (*SLC7A8*),^40^ and ATB0^+^ (*SLC6A14*).^57–59^ First, we evaluated the gene expression of these candidate transporters upon serine and glycine starvation, which we reasoned might be induced if they are involved in serine uptake. With the exception of *SLC7A8*, all of the candidate transporters are upregulated upon serine and glycine deprivation, with *SLC1A4* (which encodes the related transporter ASCT1) showing the most significant increase (Figure 3A). We also evaluated the protein expression of these transporters in ASCT2 KO cells to determine whether their expression levels might change to compensate for loss of ASCT2. Here, we found that only ASCT1 is upregulated in ASCT2 KO MCF7 cells (Figure 3B). To evaluate a role for ASCT1 in serine uptake, we generated ASCT1 and ASCT2 single and double KO MCF7 cells (Figure 3C). Importantly, while loss of ASCT1 alone slightly reduces serine uptake, loss of both transporters does not significantly reduce uptake beyond what is observed with ASCT2 alone (Figure 3E). Further, overexpression of ASCT1 to a similar degree as seen in ASCT2 KO cells induces a slight, but significant, increase in serine uptake (Figures 3F and 3G). However, combined loss of ASCT1 and ASCT2 does not dramatically reduce proliferation in low serine (Figure 3D), nor does ASCT1 overexpression rescue proliferation in low serine in ASCT2 KO cells (Figure 3H). These results suggest that ASCT1 may contribute to serine uptake in MCF7 cells, but its contribution is small relative to that of ASCT2.

To evaluate contributions to serine uptake from the other five candidate transporters, we generated single and double transporter KOs with ASCT2 (Figures S3A, S3C, S3E, S3G, and S3I), where only loss of LAT1 on its own showed a slight reduction in serine uptake, and none of the double KO cells exceeded the effects seen with ASCT2 alone (Figures S3B, S3D, S3F, S3H, and S3J). Given these results, we believe that our data are most consistent with a model in which ASCT2 is the primary serine transporter in MCF7 cells, but a network of secondary transporters can maintain serine uptake in the absence of ASCT2, particularly in high-serine conditions. Consistent with this model, all seven potential serine transporter genes are expressed in luminal breast tumors, with *SLC1A4*, *SLC38A1*, *SLC38A2*, and *SLC7A8* being enriched in luminal breast tumors relative to basal (Figures 3I–3O). However, our data also suggest that ASCT2 is likely the highest-affinity serine transporter, given that the apparent ability of other transporters to compensate for loss of ASCT2 is limited in low-serine conditions.

### Serine and glutamine compete for uptake by ASCT2

**Figure 4.**
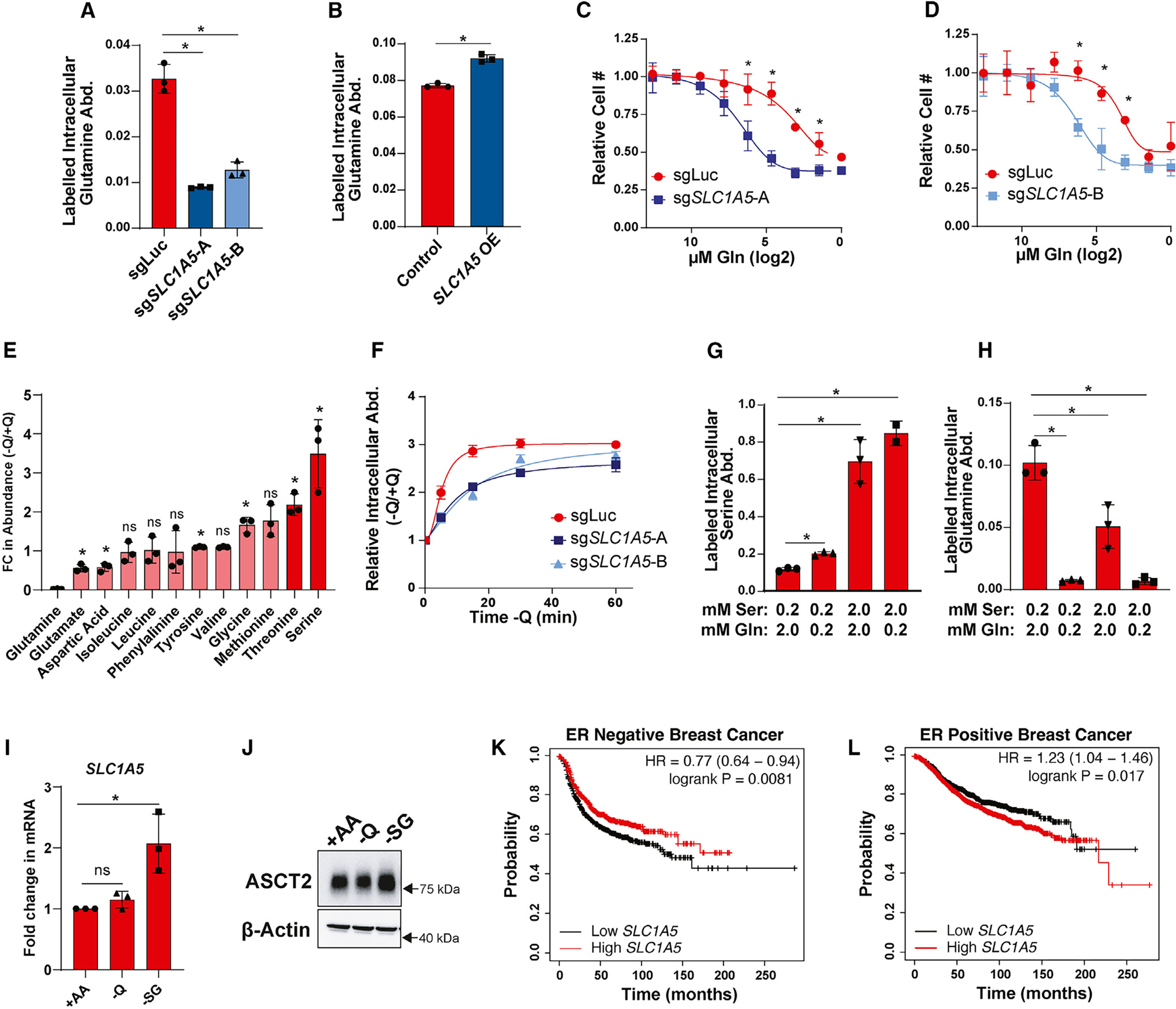
Serine and glutamine compete for uptake by ASCT2 (A) Acute glutamine uptake in MCF7 control (sgLuc) or *SLC1A5* KO (sg*SLC1A5*) cells. Values are the mean ± SD of triplicate samples from an experiment representative of three independent experiments. *p < 0.05 by Welch’s t test. (B) Acute glutamine uptake in MCF7 control or *SLC1A5* overexpressing (*SLC1A5* OE) cells. Values are the mean ± SD of triplicate samples from an experiment representative of two independent experiments. *p < 0.05 by Welch’s t test. (C and D) Glutamine dose curve with MCF7 control (sgLuc) and sg*SLC1A5* cells. Values are the mean ± SD of triplicate samples from an experiment representative of three independent experiments. *p < 0.05 by Welch’s t test. Relative cell counts are normalized to each group’s count at normal RPMI glutamine dose. (E) Intracellular amino acid abundance from MCF7 cells cultured in the absence of glutamine for 1 h. Values are the mean ± SD of the relative abundance for each amino acid in −Q relative to +Q conditions from an experiment representative of two independent experiments. FC = fold change. *p < 0.05 by Welch’s t test comparing −Q to +Q conditions. ns, not significant. (F) Intracellular serine abundance from MCF7 control (sgLuc) and *SLC1A5* KO (sg*SLC1A5*) cells cultured in the absence of glutamine for 5, 15, 30, and 60 min. Values are the mean ± SD of the relative serine abundance for each group at each time point. (G and H) Acute serine (G) and glutamine (H) uptake in MCF7 cells cultured in the indicated concentrations of serine and glutamine. Values are the mean ± SD of triplicate samples from an experiment representative of three independent experiments. *p < 0.05 by Welch’s t test. (I) qPCR data for *SLC1A5* expression in MCF7 cells cultured in complete RPMI (+AA), glutamine deprivation (−Q), or serine and glycine deprivation (−SG) for 48 h. Values are the mean ± SD of the fold change in *SLC1A5* mRNA from three independent experiments. *p < 0.05 by Welch’s t test. ns, not significant. (J) Representative western blot of MCF7 cells cultured in complete RPMI (+AA), glutamine deprivation (−Q), or serine and glycine deprivation (−SG) for 48 h. (K and L) KM Plotter survival curve results from ER-negative (K) and ER-positive (L) breast cancer patients with high or low levels of *SLC1A5* expression.

While ASCT2 has not previously been described as a serine transporter in cancer cells, it has been extensively characterized as a glutamine transporter.^47–49,60,61^ Indeed, we find that knockout and overexpression of ASCT2 in MCF7 cells causes a decrease and increase, respectively, in acute glutamine uptake (Figures 4A and 4B), and that loss of ASCT2 sensitizes MCF7 cells to decreasing glutamine concentrations (Figures 4C and 4D). To further examine ASCT2 substrate dynamics, we starved MCF7 cells of glutamine for 1 h and found that intracellular serine abundance is strongly increased upon glutamine starvation (Figure 4E). Further, we find that the accumulation of intracellular serine after glutamine starvation is delayed in ASCT2 KO cells (Figure 4F). These data are suggestive of a model where glutamine and serine compete for uptake through ASCT2. To test this model more directly, we performed serine and glutamine uptake assays in cells treated with lower or higher concentrations of serine and glutamine. Not surprisingly, we found that by raising the serine concentration to match RPMI glutamine levels (2.0 mM Ser), serine uptake is increased (Figure 4G). Interestingly, however, lowering glutamine levels by a factor of 10 (0.2 mM Gln) was also sufficient to increase serine uptake (Figure 4G), and inverting the ratio of serine to glutamine (2.0 mM Ser, 0.2 mM Gln) induces the greatest increase in serine uptake (Figure 4G). Conversely, we found that glutamine uptake is significantly reduced when either glutamine is lowered or serine is increased, or both (Figure 4H). Together, we interpret these data as being in support of a model in which serine and glutamine are competitive substrates of ASCT2. Interestingly, *SLC1A5* and ASCT2 expression are induced in response to deprivation of serine but not glutamine (Figures 4I and 4J). Further, despite a previous report suggesting a more prominent role for ASCT2-mediated glutamine uptake in triple-negative breast cancer,^47^ high expression of *SLC1A5* is associated with a decreased risk of recurrence in ER-negative breast cancer (Figure 4K), while high *SLC1A5* is associated with an increased risk of recurrence in ER-positive breast cancer (Figures 4K and 4L). Together, these results support a potential important role for ASCT2 in serine metabolism in cancer.

### ASCT2 is required for purine nucleotide biosynthesis when serine levels are limited

**Figure 5.**
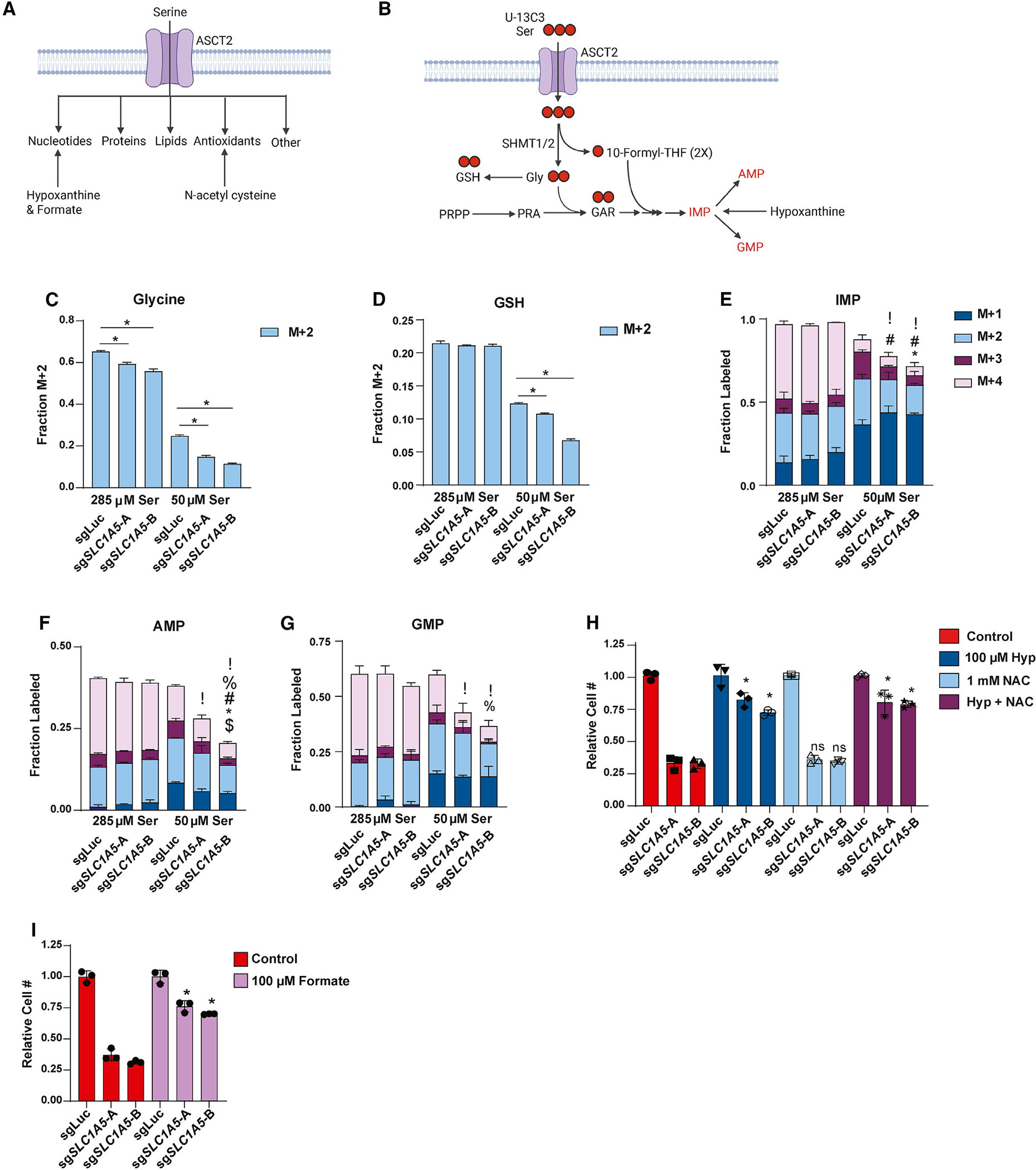
ASCT2 is required for purine nucleotide biosynthesis when serine levels are limited (A) Schematic illustrating how serine is utilized inside the cell, and how nucleotide and antioxidant metabolic pathways can be rescued in the absence of serine. (B) Schematic illustrating how U-^13^C_3_-serine can be traced through the purine and GSH biosynthesis pathways. (C–G) Stable isotope tracing results for MCF7 control (sgLuc) and *SLC1A5* KO (sg*SLC1A5*) cells cultured in media containing U-^13^C_3_-serine at normal or low serine concentrations for 8 h. Values are the mean ± SD of triplicate samples. *p* < 0.05 by Welch’s t test for each isotopomer in sg*SLC1A5* compared to sgLuc (! = M+0, % = M+1, # = M+2, * = M+3, $ = M+4). (H) Growth assay of MCF7 control (sgLuc) and *SLC1A5* KO (sg*SLC1A5*) cells cultured as indicated. Values are the mean ± SD of triplicate samples representative of three independent experiments. *p < 0.05 by Welch’s t test. ns, not significant. (I) Growth assay of MCF7 control (sgLuc) and *SLC1A5* KO (sg*SLC1A5*) cells cultured as indicated. Values are the mean ± SD of triplicate samples representative of two independent experiments. *p < 0.05 by Welch’s t test.

Serine is important for several downstream pathways (Figure 5A) and has received considerable attention for its role as a precursor for purine nucleotide and antioxidant biosynthesis.^4,62,63^ These contributions primarily occur through the action of serine hydroxymethyltransferase (SHMT1/2), which converts serine to glycine and one-carbon units that can be used in glutathione (GSH) and purine nucleotide synthesis (Figure 5B). By tracing the incorporation of serine-derived carbon (U-^13^C_3_-serine) into downstream metabolites, we observed that loss of ASCT2 results in decreased generation of glycine from serine in both complete- and low-serine conditions (Figure 5C). Moreover, while loss of ASCT2 in high serine does not strongly affect the synthesis of GSH or the purine nucleotides inosine monophosphate (IMP), adenosine monophosphate (AMP), and guanosine monophosphate (GMP), GSH and purine nucleotide biosynthesis are significantly reduced in ASCT2 KO cells in low-serine conditions (Figures 5D–5G). Importantly, because ASCT2 KO cells proliferate more slowly in low serine, the decreased biosynthesis observed in these conditions could be either a cause or a consequence of reduced proliferation. To address this, we provided cells with hypoxanthine, a substrate for the purine salvage pathway and/or the antioxidant *N*-acetylcysteine (NAC), both of which have been used previously to rescue the effects of serine starvation (Figure 5A).^19,64^ Addition of hypoxanthine was sufficient to strongly (but not completely) rescue the growth of ASCT2 KO cells cultured in low serine, while NAC had no effect either alone or in combination with hypoxanthine (Figure 5H). Importantly, both serine and glutamine from ASCT2 likely support purine nucleotide biosynthesis, and addition of hypoxanthine would be expected to rescue the contribution of both amino acids to purine biosynthesis. To isolate the contribution of serine to purine metabolism and growth, we supplemented ASCT2 KO cells with formate, which specifically supports the serine-glycine-1C pathway and not the contributions from glutamine. Here, we observed that addition of formate was sufficient to partially rescue growth of ASCT2 KO cells in low-serine conditions (Figure 5I). These results suggest that loss of ASCT2 in low serine impairs purine biosynthesis specifically due to reduced serine uptake, which results in decreased proliferation. Importantly, coordinated uptake of both serine and glutamine through ASCT2 could allow for coordinated regulation of processes that require both substrates, such as purine biosynthesis.

### ERα promotes serine uptake via direct regulation of *SLC1A5*

**Figure 6.**
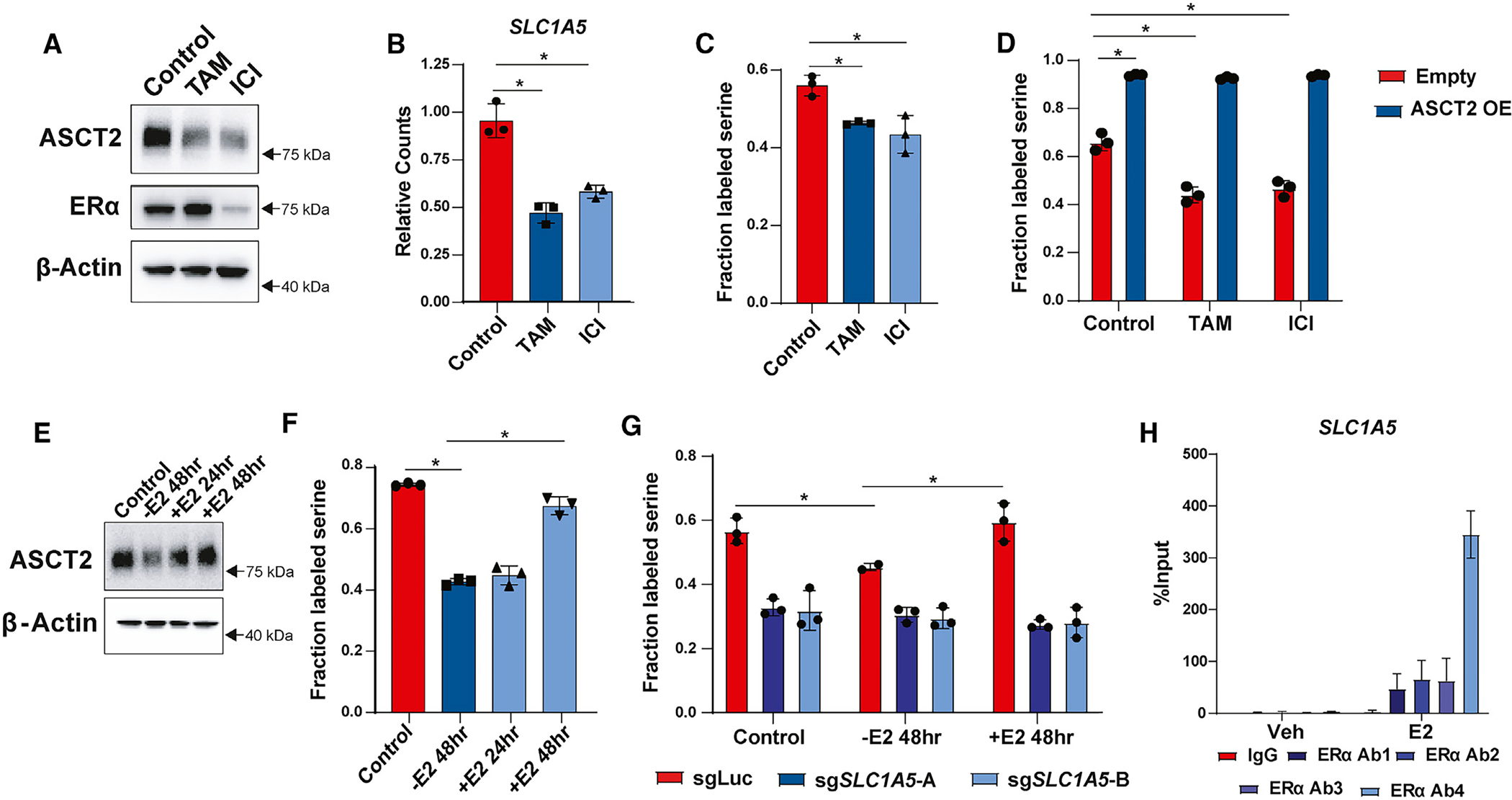
ERα promotes serine uptake via direct regulation of *SLC1A5* (A) Representative western blot of MCF7 cells cultured with DMSO (control), 1 μM tamoxifen (TAM), or 1 μM fulvestrant (ICI) for 48 h. (B) RNA-seq data from MCF7 cells cultured with DMSO (control), tamoxifen (TAM), or fulvestrant (ICI) for 48 h. Values are the mean ± SD of triplicate samples. *p < 0.05 by Welch’s t test. (C) Acute serine uptake in MCF7 cells treated with DMSO (control), tamoxifen (TAM), or fulvestrant (ICI) for 48 h. Values are the mean ± SD of triplicate values from three independent experiments. *p < 0.05 by Welch’s t test. (D) Acute serine uptake in MCF7 control and ASCT2 overexpressing (ASCT2 OE) cells treated with DMSO (control), tamoxifen (TAM), or fulvestrant (ICI) for 48 h. Values are the mean ± SD of triplicate values from two independent experiments. *p < 0.05 by Welch’s t test. (E) Representative western blot of MCF7 cells starved of estrogen for 48 h followed by add-back of 100 nM estradiol for 24 or 48 h. (F) Acute serine uptake in MCF7 cells starved of estrogen for 48 h followed by add-back of 100 nM estradiol for 24 or 48 h prior to metabolite harvest. Values are the mean ± SD of triplicate samples representative of two independent experiments. *p < 0.05 by Welch’s t test. (G) Acute serine uptake in MCF7 control (sgLuc) and *SLC1A5* KO (sg*SLC1A5*) cells under control conditions, 48 h estrogen starvation (−E2 48hr), and 48 h starvation followed by 48 h estradiol supplementation (+E2 48hr). Values are the mean ± SD of triplicate samples representative of two independent experiments. *p < 0.05 by Welch’s t test. (H) ChIP-qPCR results from MCF7 cells treated with vehicle or 10 nM estradiol for 30 min. Four unique antibodies against ERα were used along with an immunoglobulin G (IgG) control antibody. Values are the mean ± SD of triplicate samples from two independent qPCR experiments.

Most luminal breast tumors express ERα and are dependent on estrogen signaling for growth and proliferation.^65^ Because ERα is known to control many essential growth pathways, including several metabolic pathways and amino acid transporters,^66–68^ we hypothesized that estrogen signaling might also promote ASCT2 expression and serine uptake. Indeed, we found that treatment of MCF7 cells with the ERα inhibitors tamoxifen and fulvestrant (ICI 182780) lowers ASCT2 (Figure 6A) and *SLC1A5* (Figure 6B) expression and inhibits serine uptake (Figure 6C). Overexpression of ASCT2 prevents tamoxifen and fulvestrant treatment from reducing serine uptake, suggesting that the reduced serine uptake upon ERα inhibition may be due to reduced ASCT2 expression (Figure 6D). Using the EstrogeneDB database (www.estrogene.org), which compiles microarray and RNA-sequencing (RNA-seq) studies of estrogen-regulated gene expression, we observed that estrogen regulates *SLC1A5* mRNA expression in multiple ER^+^ cell lines at various doses and treatment durations (Figure S4A). Additionally, we found that estrogen starvation significantly reduces ASCT2 protein expression and serine uptake, both of which can be restimulated with 100 nM estradiol (Figures 6E and 6F). Importantly, estrogen does not regulate serine uptake in ASCT2 KO cells (Figure 6G), indicating that estrogen-dependent control of serine uptake is through regulation of ASCT2 expression. To determine whether *SLC1A5* is a direct transcriptional target of ERα, we performed chromatin immunoprecipitation (ChIP)-qPCR using four distinct ERα antibodies and found that ERα binds near the *SLC1A5* transcription start site in an estrogen-dependent manner (Figure 6H), similar to the known ERα target *EGR3* (Figure S4B). Together, our results suggest that ERα regulates serine uptake by directly promoting transcription of its bona fide target gene *SLC1A5*.

### Loss of ASCT2 induces tumor regression in combination with a serine-free diet

**Figure 7.**
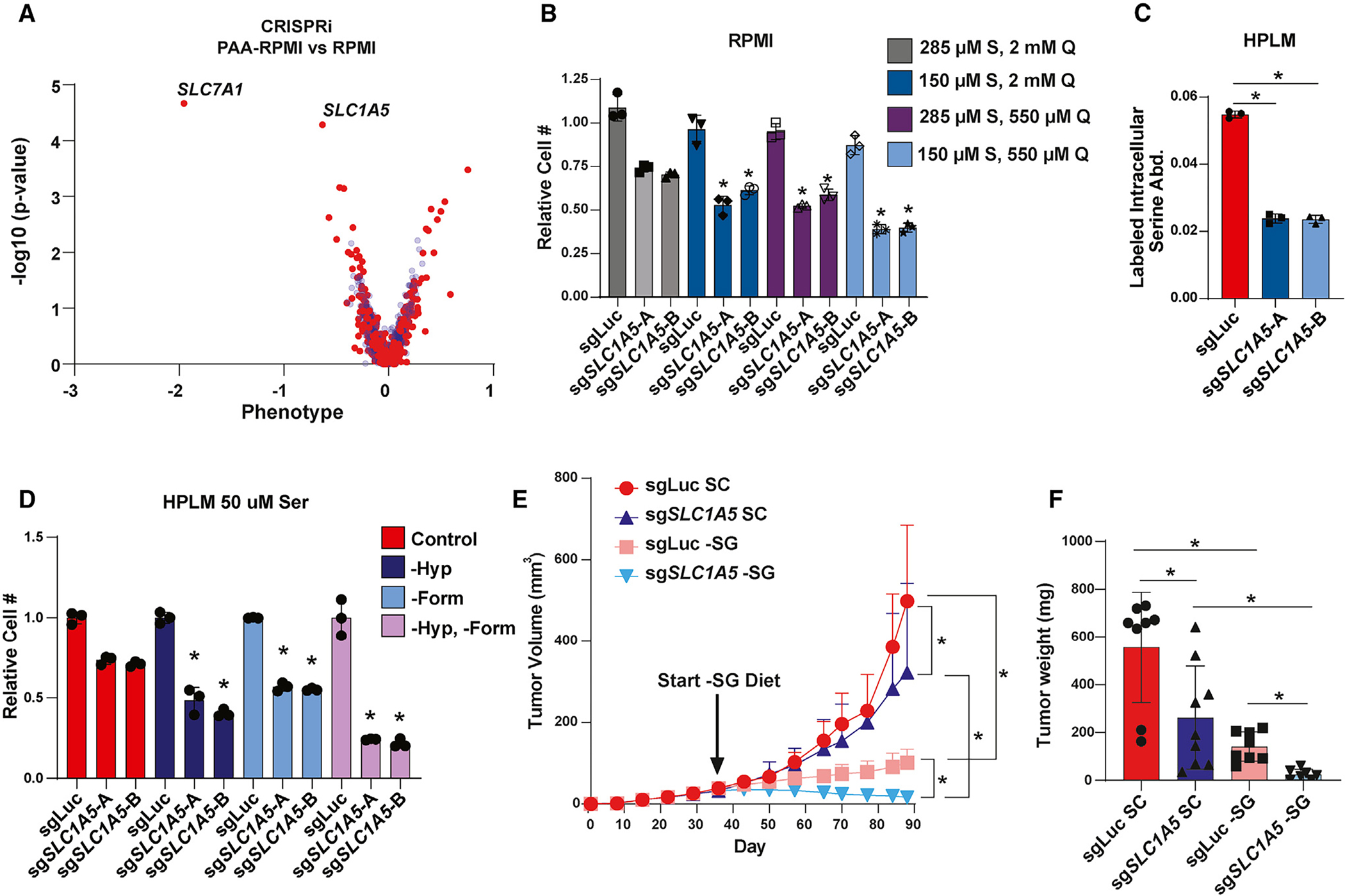
Loss of ASCT2 induces tumor regression in combination with a serine-free diet (A) CRISPRi screen results from MCF7 cells grown in RPMI compared to RPMI-PAA (physiological amino acids). Blue dots are pseudogenes that represent the technical noise of the assay, and red dots represent transporter genes. (B) Growth assay from MCF7 control (sgLuc) and ASCT2 KO (sg*SLC1A5*) cells grown in RPMI with serine and glutamine concentrations at normal RPMI or physiological levels. Values are the mean ± SD of triplicate samples representative of two independent experiments. *p < 0.05 by Welch’s t test. (C) Acute serine uptake in MCF7 control (sgLuc) and ASCT2 KO (sg*SLC1A5*) cells grown in HPLM. Values are the mean ± SD of triplicate samples from an experiment representative of three independent experiments. *p < 0.05 by Welch’s t test. (D) Growth assay from MCF7 control (sgLuc) and ASCT2 KO (sg*SLC1A5*) cells grown in low-serine HPLM (50 μM serine) in the presence or absence of 10 μM hypoxanthine and/or 50 μM formate. Values are the mean ± SD of triplicate samples from an experiment representative of two independent experiments. *p < 0.05 by Welch’s t test. (E) Tumor xenograft growth over time from MCF7 control (sgLuc) and ASCT2 KO (sg*SLC1A5*) xenografts. Mice were fed standard chow (SC) until tumors were established, then half of each cohort was switched to a serine- and glycine-free diet (−SG) for the remainder of the study. Values are the mean ± SD of each group’s tumor volume measurements. *p < 0.05 by two-way ANOVA. (F) Tumor weight at endpoint for each cohort of mice. *p < 0.05 by Welch’s t test.

Traditional cell-culture media contain supraphysiological concentrations of many amino acids.^69^ Given that extracellular serine and glutamine levels impact serine uptake (Figure 4), we assessed the activity and importance of ASCT2 in more physiological conditions. First, we performed an additional CRISPRi screen assessing transporter essentiality in MCF7 cells growing in RPMI with amino acid concentrations similar to those found in human plasma (RPMI-PAA) and compared the magnitude of transporter gene essentiality in RPMI-PAA to that in RPMI (Figure 7A). The top hit in this screen was *SLC7A1*, a known arginine transporter^70^ that is likely more essential in RPMI-PAA due to the 90% lower arginine levels found in human plasma relative to RPMI.^69^ Importantly, *SLC1A5* was the second-strongest hit that was more essential in physiological amino acid levels than in RPMI (Figure 7A). We cultured control and ASCT2 KO cells in RPMI with plasma levels of serine (150 μM), glutamine (550 μM), or both and observed increased dependence on ASCT2 in the more physiological conditions (Figure 7B), suggesting that lower serine and glutamine levels contribute to the increased *SLC1A5* essentiality in RPMI-PAA.

Next, we made use of human plasma-like medium (HPLM), which (like RPMI-PAA) contains physiological concentrations of all amino acids but also contains human plasma glucose and salt levels as well as 27 additional components found in human plasma but not RPMI or other traditional media.^69^ Notably, loss of ASCT2 decreased serine uptake in HPLM to a similar degree as seen in RPMI medium (Figure 7C), suggesting that ASCT2 is still a major serine transporter in physiological conditions. HPLM also contains 10 μM hypoxanthine and 50 μM formate, which we have found to partially rescue the growth-inhibitory effects of ASCT2 loss (Figures 5H and 5I). Indeed, removal of hypoxanthine and/or formate from HPLM strongly reduces proliferation in ASCT2 KO cells (Figure 7D). These data suggest that there are aspects of more physiological conditions that increase (higher serine/glutamine ratio) and decrease (purine/one-carbon substrates) the requirement for serine uptake mediated by ASCT2.

While HPLM can provide more physiological *in vitro* conditions, it cannot fully recapitulate the *in vivo* environment. As such, we grew control and ASCT2 KO MCF7 cells orthotopically in the mammary fat pad of nude mice and observed that loss of ASCT2 alone had only a slight inhibitory effect on tumor growth, similar to our *in vitro* results (Figures 7E and 7F). To determine whether loss of ASCT2 sensitizes to a low-serine environment *in vivo*, we switched a cohort of ASCT2 KO and control mice to a serine- and glycine-free diet (−SG) after tumors were established, whereby we observed the expected inhibitory effect of dietary serine starvation on control MCF7 tumors.^20^ Critically, we found that dietary serine starvation in addition to ASCT2 KO not only inhibited tumor growth but resulted in regression of nearly all tumors (Figures 7E and 7F). These data suggest that although ASCT2 may not be a promising therapeutic target on its own, inhibition of ASCT2 may be effective when combined with therapies that reduce serine availability *in vivo*, such as dietary serine starvation.

## DISCUSSION

In the years since serine metabolism was first implicated in cancer,^9,10^ considerable strides have been made toward understanding the central role serine plays in proliferating cells.^71^ However, despite an appreciation for the importance of exogenous serine and the potential clinical relevance of dietary serine starvation in cancer treatment,^17,72^ the transporters that mediate serine uptake in cancer cells have remained poorly characterized. In this article we identify ASCT2 (*SLC1A5*) as an important serine transporter in cancer cells. While there are numerous studies that have previously shown that ASCT2 is capable of mediating serine uptake in *in vitro* systems,^34,35,45,50^ we are the first to demonstrate this function specifically in cancer cells. Importantly, while ASCT2 is already well known in the cancer metabolism field, this is entirely due to its contributions to the uptake of glutamine rather than serine. As such, our report extends our knowledge of both serine metabolism and the function of ASCT2 in cancer.

While our original intent was to identify serine transporters in luminal/ER^+^ breast cancer cells, our studies suggest that ASCT2 may contribute to serine uptake in diverse cancer types. Importantly, however, loss of ASCT2 does not eliminate serine uptake in any model we have tested, demonstrating that there are likely one or more additional transporters that also contribute to serine uptake. Our targeted secondary transporter approach failed to identify strong contributions from ASCT1, SNAT1, SNAT2, LAT1, LAT2, or ATB0^+^ either alone or in combination with ASCT2 loss. And while the interpretation of negative data is difficult, the lack of a contribution from these transporters is notable given that all six have been shown to be capable of mediating serine uptake in *Xenopus* oocytes and/or proteoliposomes.^30–35,37,39,40,58^ This is particularly true for ATB0^+^, which has very recently been shown to contribute to serine uptake in colorectal cancer cells.^59^ This suggests that there is likely a high level of context dependence in serine uptake, where the ability to transport serine in one context does not necessarily predict transport ability in another. It is also possible that there are transporters that have not yet been shown to transport serine *in vitro* that may also support serine uptake in cancer cells. Additional work will be required to fully understand the network of serine transporters in cancer.

Because of its importance in glutamine metabolism, there have been considerable efforts to target ASCT2 in cancer.^47,60,73^ Current ASCT2 inhibitors include the pseudo-metabolites benzylserine^74^ and GPNA (L-γ-glutamyl-*p*-nitroanilide)^75^ as well as the small molecule V-9302.^76^ While benzylserine and GPNA can reduce breast cancer cell growth by inhibiting uptake of several amino acids,^74^ they will likely not be useful clinically due to the high millimolar concentrations required to be effective. Small-molecule inhibitors such as V-9302 have a higher potency and have been shown to significantly reduce glutamine uptake and cell growth in a number of cancer cell lines and xenograft models,^76^ but V-9302 likely has low specificity for ASCT2 and its effects could be due to the inhibition of multiple transporters.^77^ Nevertheless, efforts to specifically target ASCT2 are ongoing and will surely be aided by the recent publication of a complete human ASCT2 cryoelectron microscopy structure.^78,79^ Our finding that ASCT2 is an important serine transporter could expand the potential utility of ASCT2 inhibitors to include treatment of serine-auxotrophic tumors. Notably, *SLC1A5* KO mice are developmentally normal, although they do present with some immunological defects that could impact tumor immunity.^80^

Our work both *in vitro* and *in vivo* demonstrates that loss of ASCT2 has the greatest effect on cancer cell growth in low-serine conditions. This suggests that ASCT2 is the highest-affinity serine transporter expressed in these cells, while other transporters are not as efficient in promoting serine uptake in low-serine conditions. However, this conflicts with a recent report suggesting that ASCT1 has a higher affinity for serine than ASCT2.^45^ This result is not likely due to low ASCT1 expression in MCF7 cells, as direct overexpression of ASCT1 had only a minor effect on serine uptake and did not rescue cell growth in low-serine conditions. These results again highlight the likely importance of cellular context in serine uptake. Nevertheless, we find that combined loss of ASCT2 with dietary serine starvation to reduce serine levels *in vivo* has the ability to induce regression of established xenograft tumors, unlike most previous reports showing that dietary serine starvation typically only slows tumor growth. This finding may be relevant clinically, as dietary serine starvation is currently being evaluated in a clinical trial for pancreatic cancers, a subset of which is auxotrophic for serine.^23^ It is also important to note that there are environments within the body that are naturally low for serine, such as cerebrospinal fluid and brain interstitial fluid.^81–84^ While upregulation of serine biosynthesis promotes the outgrowth of triple-negative breast cancer brain metastases,^85^ it is likely that luminal breast cancer brain metastases are even more dependent on ASCT2 in this naturally low-serine environment. Therefore, further studies could identify scenarios where ASCT2 is a viable clinical target to treat serine-auxotrophic tumors.

### Limitations of the study

In this study we present data supporting a significant role for ASCT2 in promoting serine uptake in cancer cells. It is important to note, however, that this conclusion is based primarily on data from cancer cell lines growing in both traditional and physiological tissue culture media. We also performed an *in vivo* tumor xenograft experiment using MCF7 cells growing in athymic nude mice, where we observed that loss of ASCT2 had a mild inhibitory effect on tumor growth. We have previously shown that this model is highly sensitive to dietary serine starvation due to the inability to synthesize serine *de novo*. We confirmed high sensitivity to dietary serine starvation in the present study, and also found that combining dietary serine starvation with loss of ASCT2 leads to tumor regression. While this result strongly suggests that ASCT2 is also involved in serine uptake *in vivo*, additional studies will be required to confirm that ASCT2 contributes to serine uptake *in vivo*. Our *in vivo* results indicate that loss of ASCT2 alone is only sufficient to slightly reduce tumor growth, while dietary serine and glycine starvation alone has a very large effect on tumor growth. These results, along with our work *in vitro* using HPLM indicate that ASCT2 may not be a strong therapeutic target for cancer patients on its own. This, along with our working model of a potential network of serine transporters, presents potential limitations to targeting ASCT2 to treat serine-auxotrophic tumors.

## STAR★METHODS

### RESOURCE AVAILABILITY

#### Lead contact

Information or requests for reagents and resources can be directed to Jonathan Coloff (coloff@uic.edu).

#### Materials availability

Any materials generated in this study can be made available by Jonathan Coloff, lead contact, upon request.

#### Data and code availability

- Metabolomics data generated in this study is available through the NIH Common Fund’s National Metabolomics Data Repository (NMDR) Website, the Metabolomics Workbench (https://www.metabolomicsworkbench.org) where it has been assigned Study ID: ST003306. The data can be accessed directly via its Project https://doi.org/10.21228/M8VG0G. RNA sequencing data is available at NCBI GEO using the accession number GEO: GSE269606.
- This paper does not report original code.
- Any additional information required to reanalyze the data reported in this paper is available from the lead contact upon request.

### EXPERIMENTAL MODEL AND STUDY PARTICIPANT DETAILS

#### Cell culture and media

MCF7, T47D, ZR751, HCC1806 and SUM149 cells were acquired from the Brugge Lab at Harvard Medical School. A498 ccRCC cells were acquired from Frank Mason at Vanderbilt University, and A549 and Calu6 lung cancer lines were acquired from Jiyeon Kim at the University of Illinois Chicago. HCT116 colorectal cancer cell line was obtained from Nissim Hay at the University of Illinois Chicago. Cell line identity was confirmed via STR analysis. All breast cell lines were of female origin. Cell lines were tested for mycoplasma using University of Illinois at Chicago Genome Research Core facilities. Unless otherwise noted, cells were grown in RPMI with 5% dialyzed FBS (Sigma) and Pen/Strep (Invitrogen) at 37°C with 5% CO_2_. Human plasma like media (HPLM) was generated according to the published formulation^69^ with addition of 5% dialyzed FBS (Sigma) and Pen/Strep (Invitrogen). For RPMI containing altered serine or glutamine concentrations, media was formulated from RPMI powder lacking glucose and all amino acids (US Biological Life Sciences) with missing components added back as needed. Media were changed every other day for cell line propagation. For growth assays, cells were plated in complete RPMI, and medium was switched to media containing the indicated concentrations of serine and/or glutamine the next day and replenished every day for six days prior to counting with a Z1 Coulter Particle Counter (Beckman Coulter). Serine concentrations were selected based on those that were most effective for the various culture formats (e.g., flasks, multi-well plates) that all provide different cell-to-media ratios. For estrogen deprivation experiments, cells were plated in complete RPMI and allowed to attach overnight. The next day, cells were washed twice with PBS and fed RPMI lacking phenol red (Thermo Fisher Scientific) with 5% charcoal stripped FBS. Estradiol was added back to the media as needed at 100 nM. Tamoxifen (Sigma) and fulvestrant (Sigma) were added to RPMI media at 1 μM for 48hours.

#### Mouse studies

All experiments using mice were approved by the University of Illinois at Chicago Animal Care Committee. MCF7 (5 × 10^6^) sgLuc and sg*SLC1A5*-B cells were injected into the mammary fat pad at the #4 and #9 mammary glands of 6- to 8-week-old athymic nudefoxn1^nu^ female mice (Inotiv). Cell suspensions were injected at a volume of 50 μL in growth factor reduced Matrigel (Corning). Estrogen (E2) was added to the drinking water in each cage and replaced every 3–4 days. Tumor growth was measured over time via caliper measurements and tumor volume was calculated with the formula: volume = ½ (width^2^ × length). Two cohorts of mice (one sgLuc (*N* = 5) and one sg*SLC1A5*-B (*N* = 5)) were switched from standard chow to a serine and glycine free diet (Envigo, TD.160752) after palpable tumors had formed and food was replenished twice per week. At endpoint, mice were euthanized according to institutional guidelines.

### METHOD DETAILS

#### CRISPRi and CRISPRa screens

We first generated and validated MCF7 cell lines that stably express either dCas9-KRAB (MCF7 CRISPRi) or dCas9-SunTag (MCF7 CRISPRa). MCF7 CRISPRi were prepared by transduction of MCF7 cells with lentiviral particles produced using vector pMH0001 (Addgene #85969) in the presence of 8 mg/mL polybrene (Sigma). A pure polyclonal population of dCas9-KRAB expressing cells was generated by two rounds of fluorescence-activated cell sorting (FACS) gated on the top half of BFP^+^ cells (BD FACS Aria). The performance of MCF7 CRISPRi in knocking down endogenous genes was confirmed by individually targeting 3 control genes (ST3GAL4, SEL1L, DPH1) and measuring gene expression changes by RT-qPCR. To prepare MCF7 CRISPRa parental cells, MCF7 cells were first transduced with lentiviral particles produced using vector pHRdSV40-dCas9–10xGCN4_v4-P2A-BFP (Addgene #60903) in the presence of 8 mg/mL polybrene. BFP^+^ cells were isolated using one round of FACS and were subsequently transduced with lentiviral particles produced using vector pHRdSV40-scFv-GCN4-sfGFP-VP64-GB1-NLS (Addgene #60904). Cells with high GFP levels (top 25% of GFP^+^ cells) and high BFP levels (top 50% of BFP^+^ cells) were isolated by FACS and recovered in complete medium. Monoclonal cell lines were subsequently generated by limiting dilution of the sorted population in conditioned medium. Individual clones were tested for their ability to increase the expression of target control genes as previously reported.^87^ A clone exhibiting robust growth and overexpression of target genes was selected as cell line MCF7 CRISPRa.

The design and construction of transporter CRISPRi and CRISPRa sgRNA libraries has been reported elsewhere.^51^ The multiplicity of infection (MOI) of lentiviral supernatant produced from each library was determined by titration onto MCF7 CRISPRi and CRISPRa cell lines and quantification of the percentage of puromycin resistant cells. To prepare transporter MCF7 CRISPRi/a libraries, parental MCF7 CRISPRi/a cells were grown to a confluence of 80–90% in 2 × 225 cm^2^ cell culture flask (Costar) in RPMI-1640 (Corning 10–040) supplemented with 10% (v/v) heat inactivated fetal bovine serum (FBS) (Gibco 10438026) and 2.5 g/L D-Glucose. Penicillin and streptomycin were added to all screen growth media to final concentrations of 100 U/mL and 100 μg/mL, respectively (Corning 30–002–CI). Cells (about 2 × 30 million) were transduced by addition of library lentiviral supernatant in presence of 8 μg/mL polybrene to achieve an MOI of 0.3. 24 h post-transduction, cells were harvested by trypsinization (trypsin-EDTA (0.05%), Gibco 25300–054) and plated in 4 × 60 mL fresh medium in 4 × 225 cm^2^ flasks. Starting 48 h post-transduction, cells were cultured in complete medium + puromycin 1 μg/mL, and passaged as needed, for a total of 5 daily puromycin pulses. After recovery for 24 h in puromycin-free RPMI-rich (see below), library cells were harvested and homogenized, and cell pellets were resuspended at 15–20 million/mL in phosphatebuffered saline (PBS, Corning 21–040 CV). 2 × 12 million cells were washed 1× with PBS and cell pellets were stored at −80°C (T = 0 samples). Screens were initiated by addition of 9 million cells (CRISPRi) or 7 million cells (CRISPRa) to 60 mL medium in 225 cm^2^ flasks.

To prepare media for screens, we first prepared complete RPMI lacking amino acids. 8.59 g of RPMI w/o amino acids, sodium phosphate (US Biological R8999–04A), 2.00 g sodium bicarbonate (Sigma S6014), and 0.80 g sodium phosphate dibasic (Sigma S0876) were diluted in 945 mL deionized H_2_O. After addition of 100 mL dialyzed fetal bovine serum (dFBS, Gibco, Thermo Fisher Scientific 26400–044) and 5 mL D-Glucose (500 g/L), the medium was sterilized via 0.22 μm membrane filtration. Amino acids (excluding serine and glycine) were added to this base medium from stock solutions to RPMI levels. To prepare low serine medium for CRISPRi and CRISPRa screens, serine was added to this medium to 50 μM and 19 μM, respectively, (instead of 285 μM in complete RPMI). To make RPMI used as the control arm in low amino acid screens, serine and glycine were added to this medium to RPMI levels. To prepare complete RPMI medium with amino acids present at physiological levels screens (RPMI-PAA), base medium lacking amino acids was prepared as above, except that 965 mL deionized H_2_O was used. To that, amino acids were added to their concentration in human plasma using the same stock solutions as for RPMI above and as previously described.^51^ All media were pre-heated and equilibrated to 37°C/5% CO_2_ before use.

CRISPRi screens were conducted in RPMI low serine, RPMI, and RPMI-PAA in a 225 cm^2^ flask each with 2 technical replicates. Libraries were maintained at a coverage of at least 1000 × at low cell density with frequent media changes and passaging as necessary for a total of 18 days. During that time, cells in RPMI, RPMI-PAA, and RPMI low Ser underwent 8.4, 7.8, and 6.4 population doublings, respectively. CRISPRa screens were conducted in RPMI and RPMI low Ser in a 225 cm^2^ flask each with 2 technical replicates over 20 days, and maintained as above. Cells in RPMI and RPMI low Ser underwent 6.8 and 1.3 population doublings, respectively.

At screen end, 6–12 million cells were collected by centrifugation, washed 1 × with PBS and stored at −80°C. The abundance of each sgRNA in individual samples was determined as previously reported.^51^ Briefly, genomic DNA (gDNA) was extracted from cell pellets using the QIAamp DNA Blood Mini Kit (Qiagen) and typical yields ranged from 40 to 80 μg gDNA. sgRNA barcodes were amplified using 23 cycles of PCR on the gDNA from at least 6 million cells as template, Q5 polymerase (NEB M0491L) and barcoded forward and reverse primers. Amplified PCR products (~240–250 bp) were purified by agarose gel electrophoresis using the QIAquick gel extraction kit (Qiagen). Purified PCR products were quantified using the Qubit dsDNA high sensitivity assay kit (Thermo Fisher Scientific) and individual indexed libraries were mixed in equimolar ratio. Pooled libraries were sequenced on an Illumina NextSeq 500 platform using a 75 bp single read on a high output flow cell with a 2–5% PhiX spike-in. 12–20 million reads were obtained for each indexed sample. To determine counts per sgRNA sequence, reads were trimmed and aligned to the library of protospacers (93–94% of trimmed reads align). To estimate noise in the screens, simulated negative control genes (the same number as that of real transporter genes) were generated by randomly grouping 10 sgRNAs from the pool of 730 non-targeting control (NTC) sgRNAs present in the transporter libraries. For each gene (and simulated control gene), which is targeted by 10 sgRNAs, two metrics were calculated: (i) the mean of the strongest 7 rho phenotypes by absolute value (“Phenotype”), and (ii) the *p*-value of all 10 rho phenotypes compared to the 730 NTC sgRNAs (Mann-Whitney test). sgRNAs were required to have 100+ counts in at least one of the two conditions tested to be included in the analysis. To deal with noise associated with potential low count numbers, a pseudocount of 10 was added to all counts.

To determine the effect of transporter CRISPRi/a on serine transport, the two metrics were determined by comparison of sgRNA abundances in the RPMI low serine medium to those of RPMI. To determine differential transporter essentiality between RPMI-PAA and RPMI, the two metrics were determined by comparison of sgRNA abundances in RPMI-PAA to those in RPMI.

#### Western blotting

Cells were lysed in RIPA buffer (Thermo Fisher) with protease and phosphatase inhibitors (Thermo Fisher) and 1 μM MG132 (Selleckchem). Protein concentration was determined by BCA assay (Thermo Fisher). When blotting for ASCT1, SNAT1, SNAT2, LAT2, ATB0+, lysates were subject to PNGase F treatment (New England Biolabs) to deglycosylate the transporter proteins. Briefly, 20–35 μg of protein was mixed with 1 μL of Glycoprotein Denaturing Buffer (10X) and water to reach a total volume of 10 μL. The reaction was heated to 100°C for 10 min. After heating, 2 μL of GlycoBuffer 2 (10X), 2 μL of 10% NP-40, 1 μL of PNGase F and water were added to a final volume of 20 μL. The samples were allowed to incubate at 37°C for 1 h. Cell lysis samples that do not require PNGase F treatment were heated to 55°C prior to gel separation, as many transporter proteins are unstable at higher temperatures. Cell lysis protein samples were separated by gel electrophoresis on 4–20% ready-made Tris-Glycine gels (Invitrogen) and transferred at 110mV for 1 h to PVDF membranes (Millipore). Membranes were blocked with 5% milk for 1 h and incubated at 4°C overnight with one or more primary antibodies in 2% bovine serum albumin. Primary antibodies used were ASCT2 (Cell Signaling, 5245S), PHGDH (Sigma, HPA021241), PSAT1 (Thermo Fisher, PA5–22124), PSPH (Santa Cruz, sc-365183), ERα (Cell Signaling, 8644), ASCT1 (Santa Cruz, sc-393157), SNAT1 (Cell Signaling, 36057S), SNAT2 (Sigma, HPA035180), LAT1 (Cell Signaling, 5347S), LAT2 (OriGene, TA500513S), ATB0+ (US Biological, 041972), and β-Actin (Sigma, A1978). PVDF membranes were washed three times with tween 20-containing tris-buffered saline before incubation with HRP-conjugated secondary antibodies (Bio-Rad). Images were detected using a ChemiDoc MP Imaging System (Bio-Rad).

#### RT-qPCR

RNA was isolated with Trizol reagent (Thermo Fisher) and cDNA synthesis was performed using qScript cDNA synthesis Kits (QuantBio). RT-qPCR was run using SYBR Green on an ABI ViiA7 real-time PCR system (Applied Biosystems), and results were normalized to the expression of an *RPLP0* control primer set. The following primers were used: *SLC1A4* F *5*′-*GCTGTGGACTGGATTGTGG-3*′, *SLC1A4* R *5*′-*ATTCAGGTGGTGGAGAATGC-3*′, *SLC1A5* F *5*′-*CGGTCGACCATATCTCCTTG-3*′, *SLC1A5* R *5*′-*CTACATTGAGGACGGTACAGGA-3*′, *RPLPO* F *5*′-*ACGGGTACAAACGAGTCCTG-3*′, *RPLPO* R *5*′-*CGACTCTTCCTTGGCTTCAA-3’*.

#### GC-MS metabolite analyses

For serine and glutamine acute uptake assays, cells were cultured in RPMI to 80% confluency, washed with PBS and fed RPMI containing U-^13^C_3_-^15^N-serine or U-^13^C_5_-^15^N_2_-glutamine (Cambridge Isotopes) for four (serine) or seven (glutamine) minutes. Labeled media was aspirated and cells were washed with saline and then lysed with ice-cold GC-MS grade methanol (OmniSolv). Norvaline was added as an internal standard diluted in MilliQ water. GC-MS grade chloroform was added, and lysates were vortexed and centrifuged at 21,000 × g at 4°C for 10 min. The polar metabolite fraction was separated and air dried prior to derivatization with MOX (Thermo Fisher, PI45950) and *N-tert*-butyldimethylchlorosilane (TBDMS) (Sigma-Aldrich). Where necessary, labeled intracellular serine and glutamine abundance were internally normalized to the average of over 20 additional analytes that were calculated by normalizing each analyte (from a library of 20+ analytes, excluding known ASCT2 substrates serine, glutamine, and threonine) to norvaline then dividing by the average of each analyte across all samples in the batch. Because manipulation of estrogen signaling affected the abundance of numerous analytes, these experiments were analyzed as the fraction of labeled Serine 390M + 4 out of the total pool of Serine 390.

For intracellular amino acid abundance determination after glutamine deprivation, cells were lysed as described and total abundance for each amino acid was normalized to the norvaline internal standard. All samples were analyzed by GC/MS using an HP-5MS Ultra Inert GC column (19091S-433UI, Agilent Technologies) installed in an Agilent 7890B gas chromatograph coupled to an Agilent 5779B mass spectrometer. Helium was used as the carrier gas. One microliter was injected (split inlet) at 280°C. After injection, the GC oven was held at 60°C for 1 min before ramping to 320°C at 10C/min and held for 9 min at the maximum temperature. The MS system operated under electron impact ionization mode at 70 eV and the MS source and quadrupole were held at 230°C and 150°C respectively. Peak areas were determined using MassHunter software.

#### CRISPR knockout

Knockout of *SLC1A5*, *SLC1A4*, *SLC38A1*, *SLC38A2*, *SLC7A5*, *SLC7A8*, *SLC6A14*, and *PSAT1* were performed using lentiCRISPR v2 Puro or Hygro (Addgene, 52961 and 91977). The following oligos were cloned into BsmBI cut lentiCRISPR v2^86^:

*SLC1A5-A F*: *5*′-*caccgAAGAGGTCCCAAAGGCAG-3*′, *SLC1A5-A R*: *5*′-*aaacCTGCCTTTGGGACCTCTTc-3*′, *SLC1A5-B F*: *5*′-*caccgTGCCCCACAGGAAGCGGT-3*′, *SLC1A5-B R*: *5*′-*aaacACCGCTTCCTGTGGGGCAc-3*′, *SLC1A4-A F*: *5*′-*caccgAGCAGGCGTGCCAGCTGG-3*′, *SLC1A4-A R*: *5*′-*aaacCCAGCTGGCACGCCTGCTc-3*′, *SLC1A4-B F*: *5*′-*caccgCTCCGTCCATGTTCACGG-3*′, *SLC1A4-B R*: *5*′-*aaacCCGTGAACATGGACGGAGc-3*′, *SLC38A1-A F*: *5*′-*caccgATGGTGTATGAAAAGCTG-3*′, *SLC38A1-A R*: *5*′-*aaacCAGCTTTTCATACACCATc-3*′, *SLC38A1-B F*: *5*′-*caccgTTCTTCAAGAGACACAG-3*′, *SLC38A1-B R*: *5*′-*aaacCTGTGTCTCTTGAAGAAc-3*′, *SLC38A2-A F*: *5*′-*caccgATATTTGGGATATACCAG-3*′, *SLC38A2-A R*: *5*′-*aaacCTGGTATATCCCAAATATc-3*′, *SLC38A2-B F*: *5*′-*caccgAGCAGCTTCCACAGGACA-3*′, *SLC38A2-B R*: *5*′-*aaacTGTCCTGTGGAAGCTGCTc-3*′, *SLC7A5-A F*: *5*′-*caccgACGACAGCATCTGCTCGG-3*′, *SLC7A5-A R*: *5*′-*aaacCCGAGCAGATGCTGTCGTc-3*′, *SLC7A5-B F*: *5*′-*caccgTGTGGGTGGATCATGGAG-3*′, *SLC7A5-B R*: *5*′-*aaacCTCCATGATCCACCCACAc-3*′, *SLC7A8-A F*: *5*′-*caccgCATCCAACGCCGTCGCTG-3*′, *SLC7A8-A R*: *5*′-*aaacCAGCGACGGCGTTGGATGc-3*′, *SLC7A8-B F*: *5*′-*caccgTCAGGCTTCTTCCAGCGA-3*′, *SLC7A8-B R*: *5*′-*aaacTCGCTGGAAGAAGCCTGAc-3*′, *SLC6A14-A F*: *5*′-*caccgACACTCCAGAAAGAACAA-3*′, *SLC6A14-A R*: *5*′-*aaacTTGTTCTTTCTGGAGTGTc-3*′, *SLC6A14-B F*: *5*′-*caccgATATCTGACCTACAGCAA-3*′, *SLC6A14-B R*: *5*′-*aaacTTGCTGTAGGTCAGATATc-3*′, *PSAT1 F*: *5*′-*caccgACCGAGGGGCACTCTCGG-3*′, *PSAT1 R*: *5*′-*aaacCCGAGAGTGCCCCTCGGTc-3*′

#### Overexpression studies

For *SLC1A5, SLC1A4,* and *PSAT1* over expression, human *SLC1A5, SLC1A4,* and *PSAT1* were cloned into pLenti CMV Puro and *Neo* DEST, respectively (Addgene, 17452, 131962, 17392), using the gateway cloning system (Thermo Fisher). To generate lentiviral particles, HEK293T cells were transduced using PAX2, VSVG, polyethylenimine (Polysciences) and the lentiviral plasmid of interest. Viral supernatants were collected on days 2, 3, and 4 after transduction. After infection with polybrene (Sigma), cells were drug selected until mock infected cells were completely cleared, after which antibiotic was removed for further propagation.

#### ChIP-qPCR

Chromatin immunoprecipitation was performed as previously described.^88^ MCF7 cells underwent crosslinking in 1% formaldehyde in PBS. Precipitations utilized protein A Dynabeads (10003D, Invitrogen) coated with one of four ERα antibodies (Milipore 06–935, Abcam Ab3575, Cell Signaling #8644, Santa Cruz HC20). Excess antibody was washed before beginning pulldown. Samples underwent pulldown at 4°C while rotating for 16 h, after which beads were washed followed by treatment with elution buffer (0.1 M NaHCO_3_, 1% SDS) and de-crosslinking overnight at 65°C. DNA was purified using a QIAquick PCR Purification Kit followed by qPCR as described above. The following primers were used: *SLC1A5* F: *5*′-*TGCTAGCCCTGAGGCATTGT*-3′, *SLC1A5* R: 5′-*ATGCAAGCTGTCCAGGGTATT*-3’.

#### Purine biosynthesis tracing

MCF7 control (sgLuc) and AST2 KO (sg*SLC1A5*) cells were plated in triplicate and allowed to attach overnight. The next day, cells were washed with PBS and fed U-^13^C-serine at complete RPMI (285 μM) or low (50 μM) concentrations for 8 h. After incubation with labeled serine media, 1mL of media from each well was collected to be stored at −20°C and the remaining media was discarded. Cells were washed with 1mL of ice-cold PBS on ice followed by 500 μL of 80% LC/MS-grade methanol (OmniSolv). The cells were then incubated at −80°C for 30 min. Each well was scraped, and the lysate-methanol mixture was transferred to 1.5mL Eppendorf tubes, which were centrifuged at 17,000 × *g* for 20 min at 4°C. Supernatant was collected from each tube and stored at −80°C until LC/MS analysis.

U-^13^C_3_-Serine labeled samples were analyzed using the Vanquish UPLC system coupled to a Q Exactive HF (QE-HF) mass spectrometer equipped with HESI (Thermo Fisher). The instrument conditions were optimized based on established methods.^89^ 5 μL of all samples were injected by an auto-sampler and separated by an Atlantis Premier BEH Z-HILIC VanGuard FIT column (2.1 mm × 150 mm, 2.5 μm (Waters)) using a gradient elution of water containing 10mM ammonium carbonate and 0.05% ammonium hydroxide (Solvent A) and acetonitrile (Solvent B) at a flow rate of 0.25 mL/min. Injected samples were eluted by linear gradient from 80% to 20% Solvent B in 13 min. This percentage was maintained for 2 min and equilibrium time of 4.9 min. The eluted metabolites were detected using negative electrospray ionization mode. Full MS spectra were captured from 65 to 975 m/z at 120,000 resolutions with an automatic gain control (AGC) set to 3×10^6^ charges. The capillary temperature and voltage were 320°C and 3.5kV, respectively.

Following the initial conversion of the.raw data files to the.cdf format using Xcalibur (Version 4.0), we performed further data processing to facilitate targeted metabolomics analysis. This subsequent processing was carried out utilizing El-Maven (Version 0.10.0 or 0.12.0), with default parameters maintained except: the ionization mode was set to negative, an isotopic tracer of C13 was employed, and the extracted-ion chromatogram (EIC) extraction window was established at ±15.00 ppm. Metabolite identification relied on matching retention time and precursor ion m/z with our established library.^90^ Peak intensity in EIC was measured using AreaTop. Isotope correction utilized El-Maven’s output in IsoCor (Version 1.0 or 2.2.0), with parameters: 13C tracer, ‘Low resolution’, ‘Correct natural abundance’ selected; isotopic purity: 12C (0.01), 13C (0.99).

#### RNA-sequencing

MCF7 cells were treated with DMSO vehicle control, tamoxifen (1 μM), or fulvestrant (1 μM) in HPLM with or without serine and glycine (SG), with 5% dialyzed FBS (Sigma) and Pen/Strep (Invitrogen). After 48 h of treatment, RNA was isolated from the cells using Trizol reagent (Thermo Fisher). RNA samples were sent to Novogene for human mRNA sequencing using Illumina NovaSeq 6000 platform and paired end 150 bp (PE150) sequencing strategy. This service included library preparation prior to sequencing and quantification analysis.

#### Charcoal stripped serum

A charcoal-dextran solution (5 mg of charcoal (Fisher), 500 mg dextran (Sigma), 50 mL 0.15 M NaCl (Fisher)) was added to a 500 mL bottle of FBS (Sigma) and the mixture was incubated for 45 min at 56°C with 180 rpm rotation. After shaking, the mixture was centrifuged at 4000 rpm for 20 min at room temperature. An additional 25 mL of charcoal-dextran solution was added and the same rotation/incubation and centrifugation was performed two additional times. The mixture was then sterile-filtered through a 0.45 μm bottle-top filter and the filtered again through a 0.22 μm bottle-top filter. Charcoal-stripped FBS was aliquoted and stored at −20°C.

### QUANTIFICATION AND STATISTICAL ANALYSIS

The transporter-specific CRISPR screen compares the individual transporter gene average of the strongest 7 phenotype scores from the 10 sgRNAs used for each gene with the *p*-value of all 10 phenotypes compared to the 730 non-targeting control gene sgRNAs (Mann-Whitney test). The results displayed as volcano plots represent a single score calculated by multiplying the phenotype score with the −log10(*p*-value). Only sgRNAs with a count greater than 100 in at least one of the media conditions were included in the final analysis. Volcano plots were generated in GraphPad Prism. All other statistical tests (as indicated in the figure legends) were performed using GraphPad Prism. Kaplan-Meier plots were generated using KM Plotter using the auto select best cutoff feature.^91^

## Supplementary Material

1

2

3

## Figures and Tables

**KEY RESOURCES TABLE:** 

REAGENT or RESOURCE	SOURCE	IDENTIFIER

Antibodies		
ASCT2	Cell Signaling	5245S
PHGDH	Sigma	HPA021241
PSAT1	Thermo Fisher	PA5–22124
PSPH	Santa Cruz Biotechnology	sc-365183
ERα	Cell Signaling	8644
ASCT1	Santa Cruz Biotechnology	sc-393157
SNAT1	Cell Signaling	36057S
SNAT2	Sigma	HPA035180
LAT1	Cell Signaling	5347S
LAT2	OriGene	TA500513S
ATB0+	US Biological	041972
β-Actin	Sigma	A1978
Bacterial and virus strains		
NEB Stable Competent *E. coli* (High Efficiency)	New England Biolabs	C3040I

Chemicals, peptides, and recombinant proteins		
MOX	Thermo Fisher	PI45950
TBDMS (N-*tert*-butyldimethylsilyl-N-methyltrifluoroacetamide with 1% tert-butyldimethylchlorosilane)	Sigma	375934
Experimental models: Cell lines		
MCF7	Brugge Lab	N/A
T47D	Brugge Lab	N/A
ZR751	Brugge Lab	N/A
HCC1806	Brugge Lab	N/A
SUM149	Brugge Lab	N/A
A498	Mason Lab	N/A
A549	Kim Lab	N/A
Calu6	Kim Lab	N/A
HCT116	Hay Lab	N/A
Experimental models: Organisms/strains		
Athymic nude-foxn1^nu^ female mice	Inotiv	N/A
Control diet	Envigo	TD.110839
Serine and glycine free diet	Envigo	TD.160752
Oligonucleotides		
Primers for *SLC1A4, SLC1A5*, and RPLPO (see ChIP-qPCR section of STAR Methods)	This paper	N/A
Oligos to generate sgRNAs targeting *SLC1A5, SLC1A4, SLC38A1, SLC38A2, SLC7A5, SLC7A8, SLC6A14*, and *PSAT1* (see CRISPR Knockout section of STAR Methods)	Sequences from (Park et al.^86^)	Park et al.^86^
Primers for *SLC1A5* (see chip-qpcr section of STAR Methods) This paper		N/A
Recombinant DNA		
lentiCRISPR v2 Puro	Addgene	52961
lentiCRISPR v2 Hygro	Addgene	91977
pLenti CMV *Neo*	Addgene	17392
pLenti CMV Puro	Addgene	17452
Deposited data		
Metabolite abundance data	NMDR	ST003306
RNA sequencing data	NCBI GEO	GSE269606

## Highlights

- ASCT2 contributes to serine uptake in cancer cells
- Serine and glutamine compete for uptake via ASCT2
- ERα promotes serine uptake by directly activating *SLC1A5* transcription
- Loss of ASCT2 combined with dietary serine starvation shrinks xenograft tumors
