# Supplementary material for: ASCT2 is a major contributor to serine uptake in cancer cells
Source: Cell Rep. Author manuscript; Available in PMC 2024 Sep 17. (PMC11406281; doi:10.1016/j.celrep.2024.114552)
Supplement: 1 [file NIHMS2019524-supplement-1.pdf]

Cell Reports, Volume *43*

Supplemental information

ASCT2 is a major contributor to serine uptake in cancer cells

Kelly O. Conger, Christopher Chidley, Mete Emir Ozgurses, Huiping Zhao, Yumi Kim, Svetlana E. Semina, Philippa Burns, Vipin Rawat, Lina Lietuvninkas, Ryan Sheldon, Issam Ben-Sahra, Jonna Frasor, Peter K. Sorger, Gina M. DeNicola, and Jonathan L. Coloff

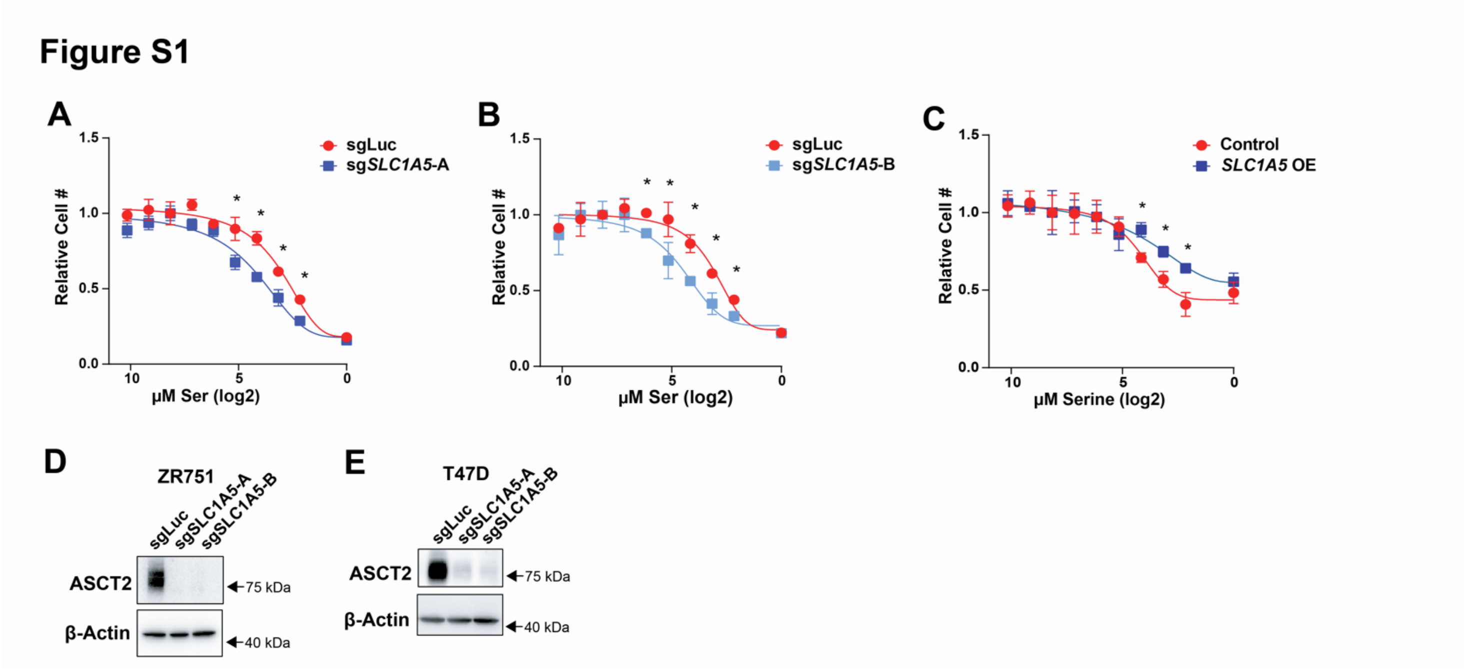

Figure S1: Identification of ASCT2 as a serine transporter in luminal breast cancer cells

A-B) Serine dose curves with MCF7 control (sgLuc) and ASCT2 KO (sg*SLC1A5*) cells. Values are the means ±SD of triplicate samples from a representative experiment of 3 independent experiments. *$p < 0.05$ by Welch's t-test. Cell numbers normalized to each group's count at normal RPMI serine dose.

C). Serine dose growth curve with MCF7 control and ASCT2 over-expressing (*SLC1A5* OE) cells. Values are the means ±SD of triplicate samples from an experiment representative of 3 independent experiments. *$p < 0.05$ by Welch's t-test comparing individual serine doses. Cell numbers normalized to each group's count at normal RPMI serine dose.

D). Representative western blot from ZR751 control (sgLuc) and ASCT2 KO (sg*SLC1A5*) cells.

E). Representative western blot from T47D control (sgLuc) and ASCT2 KO (sg*SLC1A5*) cells.

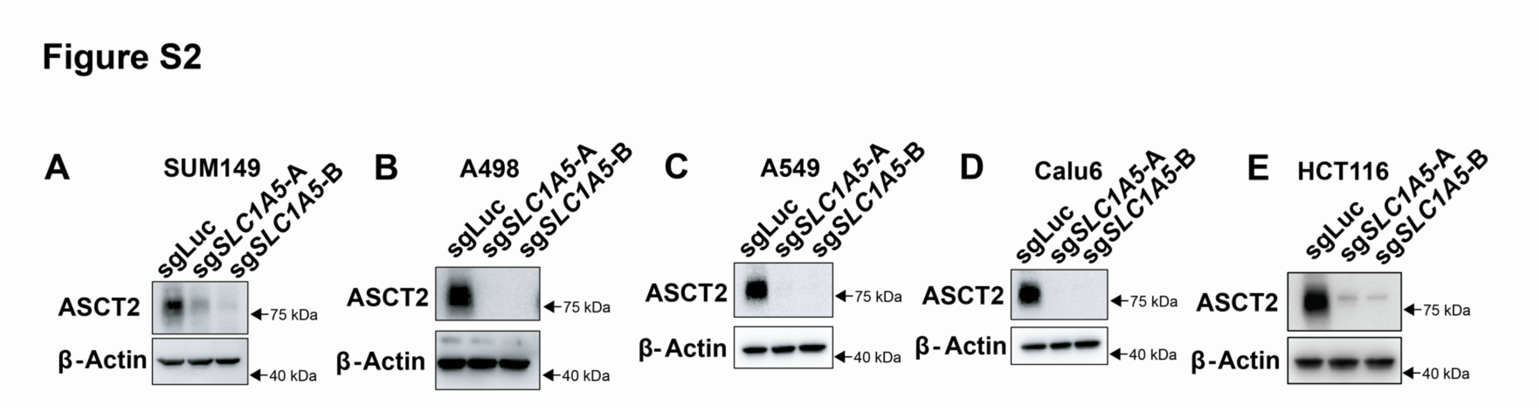

Figure S2. ASCT2 contributes to serine uptake in non-auxotrophic cells

A-E). Representative western blot from SUM149 (A), A498 (B), A549 (C), Calu6 (D) and HCT116 (E) control (sgLuc) and ASCT2 KO (sg*SLC1A5*) cells.

Figure S3

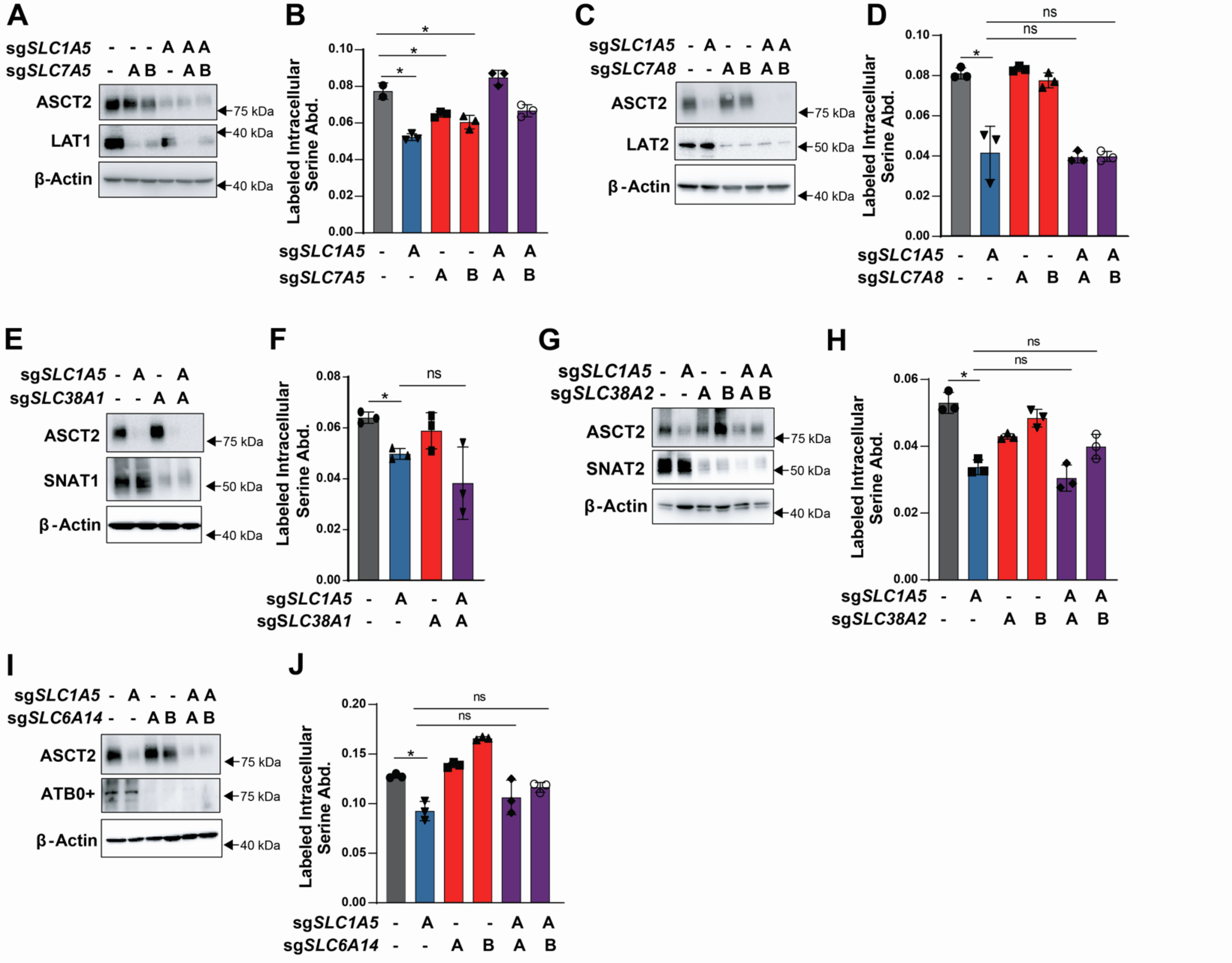

Figure S3. Transporter compensation in the absence of ASCT2

A). Western blot from single and double LAT1 (sg*SLC7A5*) and ASCT2 (sg*SLC1A5*) KO MCF7 cells.

B). Acute serine uptake for single and double LAT1 (sg*SLC7A5*) and ASCT2 (sg*SLC1A5*) KO MCF7 cells. Values are the mean ±SD of triplicate samples from an experiment representative of 2 independent experiments. *p < 0.05 by Welch's t-test.

C). Western bot from single and double LAT2 (sg*SLC7A8*) and ASCT2 (sg*SLC1A5*) KO MCF7 cells.

D). Acute serine uptake for single and double LAT2 (sg*SLC7A8*) and ASCT2 (sg*SLC1A5*) KO MCF7 cells. Values are the mean ±SD of triplicate samples from an experiment representative of 2 independent experiments. *p < 0.05 by Welch's t-test.

E). Western blot from single and double SNAT1 (sg*SLC38A1*) and ASCT2 (sg*SLC1A5*) KO MCF7 cells.

F). Acute serine uptake for single and double SNAT1 (sg*SLC38A1*) and ASCT2 (sg*SLC1A5*) KO MCF7 cells. Values are the mean ±SD of triplicate samples from an experiment representative of 3 independent experiments. *p < 0.05 by Welch's t-test.

G). Western blot for single and double SNAT2 (sg*SLC38A2*) and ASCT2 (sg*SLC1A5*) KO MCF7 cells.
H). Acute serine uptake for single and double SNAT2 (sg*SLC38A2*) and ASCT2 (sg*SLC1A5*) KO MCF7 cells. Values are the mean ±SD of triplicate samples from an experiment representative of 3 independent experiments. *$p < 0.05$ by Welch's t-test.
I). Western blot for single and double ATB0+ (sg*SLC6A14*) and ASCT2 (sg*SLC1A5*) KO MCF7 cells.
J). Acute serine uptake for single and double ATB0+ (sg*SLC6A14*) and ASCT2 (sg*SLC1A5*) KO MCF7 cells. Values are the mean ±SD of triplicate samples from an experiment representative of 3 independent experiments. *$p < 0.05$ by Welch's t-test.

Figure S4

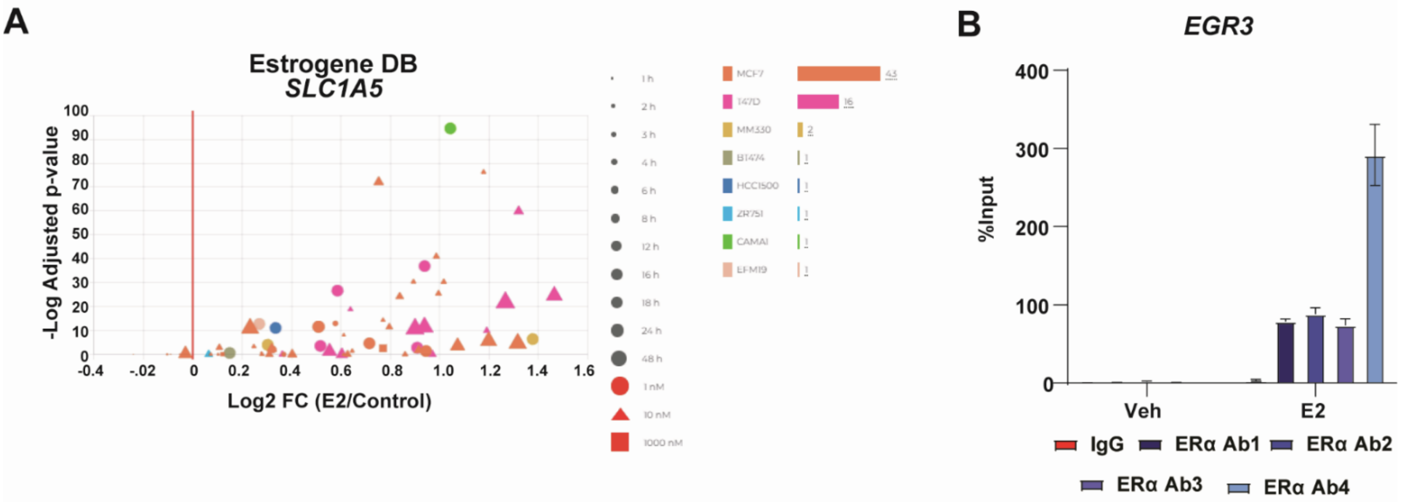

Figure S4. ERα promotes serine uptake via direct regulation of *SLC1A5*

A). RNA-Seq Analysis of *SLC1A5* expression after stimulation with different doses of estradiol in breast cancer cell lines from Estrogene DB (www.estrogene.org).
B). ChIP-qPCR results from MCF7 cells treated with vehicle or 10 nM estradiol for 30 minutes. Four unique antibodies against ERα were used along with an IgG control antibody. Values are the means ±SD of triplicate samples from two independent qPCR experiments. *EGR3* is a known transcriptional target of ERα and therefore acts as a positive control.
